# Host range of strand-biased circularizing integrative elements: a new class of mobile DNA elements nesting in *Gammaproteobacteria*

**DOI:** 10.1186/s13100-023-00295-5

**Published:** 2023-05-26

**Authors:** Desmila Idola, Hiroshi Mori, Yuji Nagata, Lisa Nonaka, Hirokazu Yano

**Affiliations:** 1 Graduate School of Life Sciences, Tohoku University, 2-1-1 Katahira, Aobaku, Sendai, 980-8577 Japan; 2 Department of Informatics, National Institute of Genetics, 1111 Yata, Mishima, 411-8540 Japan; 3 Faculty of Human Life Sciences, Shokei University, 2-6-78 Kuhonji, Kumamoto, 862-8678 Japan; 4 Antimicrobial Resistance Research Center, National Institute of Infectious Diseases, 4-2-1 Aobacho, Higashimurayama, Tokyo, 189-0002 Japan

**Keywords:** Integrase, SE, GInt, RIT, ICE, IME, Copy-out, MexCD-OprJ, *mcr-9*, Transposon

## Abstract

**Background:**

The strand-biased circularizing integrative elements (SEs) are putatively non-mobilizable integrative elements for transmitting antimicrobial resistance genes. The transposition mode and the prevalence of SEs in prokaryotes remain vague.

**Results:**

To corroborate the transposition mode and the prevalence of SEs, hypothetical transposition intermediates of an SE were searched for in genomic DNA fractions of an SE host. Then, the SE core genes were defined based on gene knockout experiments, and the synteny blocks of their distant homologs were searched for in the RefSeq complete genome sequence database using PSI-BLAST. A genomic DNA fractionation experiment revealed that SE copies are present in a double-stranded nicked circular form in vivo. Operonic structure of three conserved coding sequences (*intA*, *tfp, intB*) and *srap* located at the left end of SEs were identified as essential for *attL* × *attR* recombination. The synteny blocks of *tfp* and *srap* homologs were detected in 3.6% of the replicons of *Gammaproteobacteria* but not in other taxa, implying that SE movement is host-dependent. SEs have been discovered most frequently in the orders *Vibrionales* (19% of replicons), *Pseudomonadales* (18%), *Alteromonadales* (17%), and *Aeromonadales* (12%)*.* Genomic comparisons revealed 35 new SE members with identifiable termini. SEs are present at 1 to 2 copies per replicon and have a median length of 15.7 kb. Three newly identified SE members carry antimicrobial resistance genes, like *tmexCD-toprJ*, *mcr-9*, and *bla*_GMA-1_*.* Further experiments validated that three new SE members possess the strand-biased *attL* × *attR* recombination activity.

**Conclusions:**

This study suggested that transposition intermediates of SEs are double-stranded circular DNA. The main hosts of SEs are a subset of free-living *Gammaproteobacteria*; this represents a rather narrow host range compared to those of mobile DNA element groups discovered to date. As the host range, genetic organization, and movements are unique among the mobile DNA elements, SEs provide a new model system for host-mobile DNA element coevolution studies.

**Supplementary Information:**

The online version contains supplementary material available at 10.1186/s13100-023-00295-5.

## Background

Transposons are specific DNA segments that can repeatedly move from one location to another in one or more genomes [1]. Although the mutagenic activity of transposons might have both positive and negative impacts on cellular fitness [2, 3], the presence of a few transposon copies in a genome is thought to drive the population’s evolution [4]. In microorganisms where horizontal gene transfer occurs easily, DNA strand exchange activities of transposons can lead to gene capture, reordering, and formation of plasmid fusions, accelerating the adaptation of microbial populations to many antimicrobials used by humans [5–8].

The integrative and conjugative elements (ICEs), a class of mobile DNA elements, move from one location in one genome to a few selected locations in other genomes using site-specific recombinase and conjugation machinery [9]. ICEs follow multiple steps for movement: excision of the double-stranded ICE DNA as a circular molecule, conjugative transfer, and integration of double-stranded ICE DNA (classified as ‘cut-out paste-in’ movement [10]). There are ICE-like integrative elements that lack conjugation-associated genes, like IME, CIME, MGI, MTn, and IE (hereafter, collectively IMEs) [11–15]. The excision of these known ICE/IMEs generates empty donor sites [14, 16, 17].

**Fig. 1:**
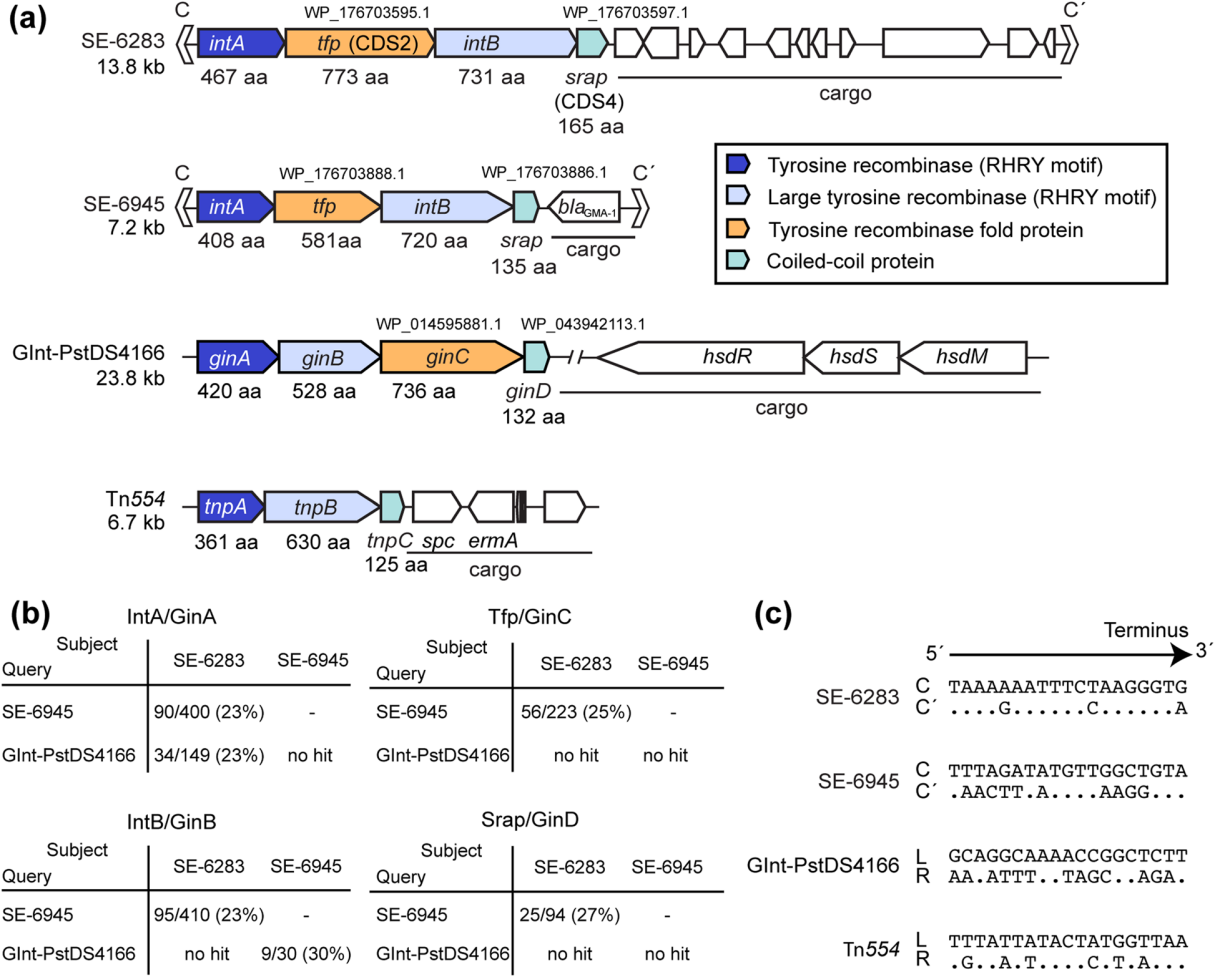
**a** Genetic organization of the known SEs, GInt and Tn*554*. *IntA*, *intB, ginA, ginB, tnpA*, and *tnpB* products contains the RHRY motif of tyrosine recombinase. GInt from *P. stuzeri* strain DSM 4166 chromosome is here referred to as GInt-PstDS4166. *Tfp*, and *ginC* products are predicted to have a tyrosine recombinase core site-binding domain in the center of the molecule but lack the catalytic RHRY motif. The locus_tags of SE core genes in GenBank accession number AP024165.1 are as follows: *intA*_SE-6283_, VYA_19760; *tfp*_SE-6283_, VYA_19770; *intB*_SE-6283_, VYA_19780; *srap*_SE-6283_, VYA_19790; *intA*_SE-6945_, VYA_04400; *tfp*_SE-6945_, VYA_04410; *intB*_SE-6945_, VYA_04420; *srap*_SE-6945_, VYA_04430. Information of Tn*554* sequence is from GenBank accession no. X03216. **b** Identity between homologs based on blastp. No hit indicates the hit sequence with E-value < 0.5 was not detected. **c** Terminal sequences of SEs and GInt. Dot in motif C′ indicates that the nucleotide is identical to that in C. Unnamed terminal sequences of GInt and Tn*554* are labelled L and R for left and right ends, respectively

**Fig. 2:**
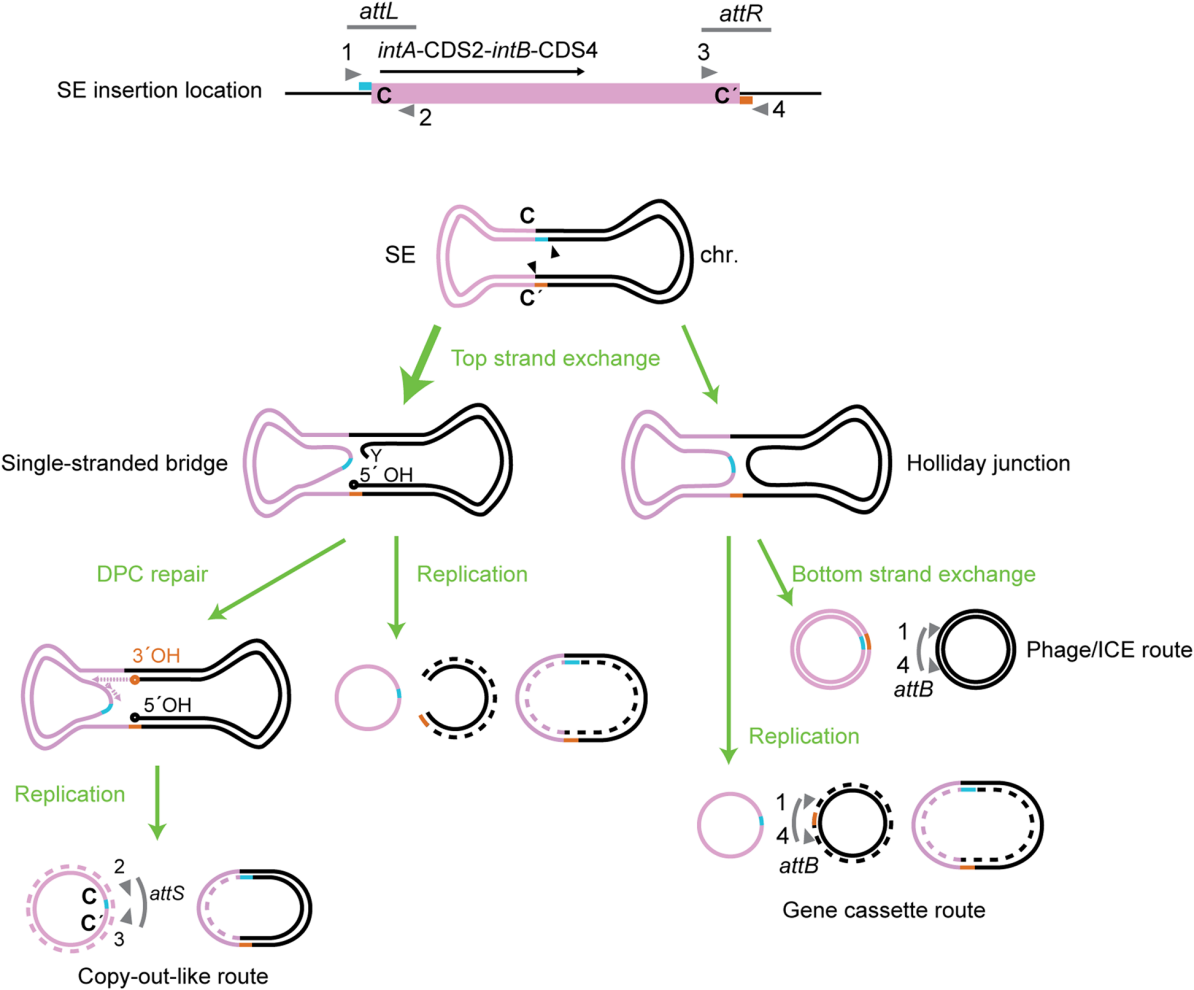
Models for the formation of SE circular transposition intermediates. Upper panel: Schematic of SE structure. The *intA*-proximal border between SE and the insertion location is termed *attL*, while *intA*-distal border is termed *attR* [18]. The triangle in gray, with number 1, 2, 3, or 4, indicates the primer annealing position used for PCR detection of *attL*, *attR*, *attS*, and *attB* throughout this study. Lower panel: four hypothetical routes to circular transposition intermediate formation. The most likely path suggested by previous studies [18, 19] is indicated by the thicker arrow. Six bases next to C and C′ are shown in distinct colors (cyan/orange) to easily track their fates in the strand exchange processes. The triangle in black indicates nicking at the top strand. Left: the most likely model for SE’s route. A single-stranded DNA bridge is generated by top strand exchange in *attL* ×﻿ *attR* recombination, leaving the host DNA side unjointed. Cleaning of the covalently linked integrase by DNA–protein crosslink (DPC) repair allows replication, resulting in the IS*911-*like copy-out [21]. Right: Phage/ICE/gene cassette routes (unlikely for SEs). Top strand exchange generates a Holliday junction (HJ). Bottom strand exchange generates circular DNAs with a heteroduplex spacer like Tn*916* excision [22]. Replication of the HJ-forming molecule generates a single-strand gene cassette-like circle and a molecule with *attB*

A new group of mobile DNA elements, called strand-biased circularizing integrative elements (SEs), have recently been identified as transposable elements inserted into *E. coli*’s chromosome during mating between *Vibrio alfacsensis* and *E. coli* [18, 19]. SE-6283 is a 13.8 kb element carrying no identifiable phenotypic marker gene, while SE-6945 is a 7.2 kb element carrying *bla*_GMA-1_ encoding class A β-lactamase (Fig. 1a). The two elements are located in both the chromosome and conjugative multidrug resistance plasmid pSEA1 of *Vibrio alfacsensis* marine isolate 04Ya108 [19]. SEs have four conserved coding sequences, *intA*, CDS2, *intB*, and CDS4, between the 13 to 19 bp imperfect inverted repeats (Fig. 1a). Both *intA* and *intB* encode tyrosine recombinases that possess a catalytic RHRY motif [19, 20], whereas the products of coding sequences CDS2 and CDS4 are hypothetical proteins. SEs possess five unique features: (i) once integrated into a target site (*attB*), an empty site is not generated despite occurrence of the left border (*attL*) × right border (*attR*) recombination; (ii) 6 bases next to the motif C end, but not the motif C′ end, are preferentially incorporated to the *attL* × *attR* recombination products, thus the transposition intermediate of SE is hypothesized to be a single-stranded circle of one specific strand (top strand in Fig. 1a) or its replicated double-stranded form (Fig. 2); (iii) when inserting into a target site, the 6 bp (or 6 base) spacer between the motifs C and C′ at the joint region on the putative circular SE (termed *attS*) is preferentially placed next to motif C′ in newly formed *attR*, whereas the central 6 bp in *attB* is placed next to motif C in *attL* [18, 19], thus 6 bp next to motif C in *attL* and motif C′ in *attR* behaves as ‘fingerprint’ and ‘footprint’ respectively of the SEs; (iv) the entire excision(copy-out-like)/integration(copy-in-like) process must be catalyzed by the tyrosine recombinases since no other type of enzyme which catalyzes DNA strand nicking and strand transfer (such as an HUH endonuclease and a DDE transposase) is encoded by the SEs and (v) the SEs integrate into a few selected locations in the genome.

Bardaji et al*.* [23] reported SE-like genomic islands, as GInts (Genomic Islands with three Integrases) (Fig. 1a), in the genomes of the plant-associated *Pseudomonas* species and several other taxa, independent of SEs. GInts carry four conserved coding sequences (*ginA, ginB, ginC,* and *ginD*), three of which encode tyrosine recombinase or its related protein. This genetic structure is reminiscent of putative mobile DNA elements, termed RIT (Recombinase In Trio) elements discovered in diverse taxa [24–26]. Bardaji et al*.* demonstrated all four coding sequences to be essential for both *attL* × *attR* recombination and the integration of synthetic circular GInt into the chromosomal target site in vivo [23]. However, transposition of the full-length GInt has not yet been demonstrated. In three of the seven GInt + strains analyzed, GInt did not generate an empty site after *attL* ×﻿ *attR* recombination, which is a characteristic SE phenomenon not observed in ICE excision. However, potential strand bias in the GInt-associated recombination remains elusive. The blastn- or blastp-based searches have failed to detect a similarity between the SE and GInt genes (Fig. 1b). GInts are also putatively non-mobilizable [23].

Key SE features include: the implication of one ordinary and one large tyrosine recombinases with RHRY motif, the presence of target specificity, the apparent lack of mobilization system, and a 6-bp footprint. These features are shared with Tn*554* discovered from genera *Staphylococcus* and *Enterococcus* of phylum *Firmicutes* [27–30]. CDS2’s counterpart is lacking in Tn*554*, while CDS4’s gene product shows similarity to TnpC of Tn*554* on the secondary structure level (Fig. 1a). Terminal sequences of Tn*554* form imperfect inverted repeats (Fig. 1c). Whether the *attL* × *attR* recombination-equivalent process of Tn*554* generates an empty site, and whether it generates a circular transposition intermediate has not been evaluated to date. Hence, the transposition route of Tn*554* has yet not been resolved.

Transposons in prokaryotes, including ICEs/IEs, are expected to have a wide host range because the DNA strand nicking/exchange process itself requires only one or a few proteins, such as DDE transposase alone [31] or Int plus Xis [16, 32–34]. In fact, Tn*3* family transposons have been discovered in both Gram-positive and Gram-negative bacteria [35]. Members of ICEs have been discovered in the archaea and bacteria [36]. There are only three relevant pieces of literature on the movements of SEs and GInts [18, 19, 23]. SEs/GInts have thus been hypothesized to be present in limited prokaryotic taxa and most of them are predicted to remain inactive under normal physiological conditions. Therefore, identification of undiscovered SEs and their corresponding hosts would contribute to expanding our understanding of prokaryotic genomic organization, particularly about the unknown roles of certain genomic regions. Identification of novel SEs will also improve our understanding of the fundamental process of how mobile element families emerged in Earth’s evolutionary history. Therefore, this study aimed to discover new SEs through database searches and determine their host ranges by quantitating the discovery rate of SE per taxon.

As experimental evidence indicative of a copy-out-like transposition route of SEs/GInts are still poor in the existing literature, in this study we began by obtaining additional evidence for the presence of a hypothetical circular transposition intermediate of an SE in the genomic DNA. Then, the essential nature of conserved SE genes in *attL ×* *attR* recombination was evaluated using gene knockout experiments. Furthermore, based on the database survey focusing on two proteins unique to SEs, we show SEs to be active in transmitting and diversifying in the extant bacteria belonging to phylum *Gammaproteobacteria* (also called *Pseudomonadota*), particularly in the genera *Vibrio, Shewanella, Laclercia*, *Alteromonas*, and *Pseudomonas.*

## Results

### Transposition intermediates of SEs are composed of double-stranded nicked DNA forms

The current model for the SE transposition route is to pass the figure-eight structure containing a single-stranded bridge because the empty site is not generated despite occurrence of *attL ×* *attR* recombination in the case of two known transposable SEs (Fig. 2) [18, 19]. Replication of the figure-eight structure can produce either single-stranded SE circles or double-stranded SE circles as transposition intermediates of SEs (Fig. 2); however, hypothetical circular transpositional intermediates in vivo had not previously been captured by experimental observation.

**Fig. 3:**
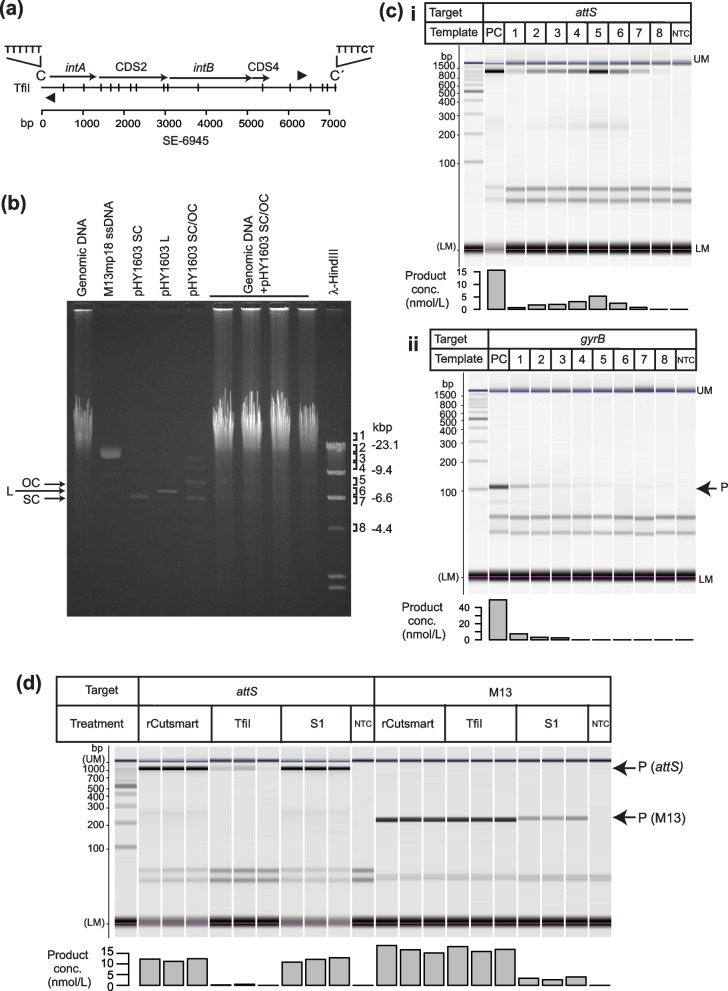
PCR detection of *attS* in fractions of genomic DNA. **a** Restriction map of SE-6945 located on the chromosome 1 of *V. alfacsensis* strain 04Ya108 (Genbank accession no. AP024168.1). The positions of TfiI sites are indicated by vertical lines. Filled arrowheads indicate the annealing positions of outward-facing primers, 6945_L_out, and 6945_R_out. **b** Agarose gel electrophoresis of BHY606 genomic DNA with the circular SE analog pHY1603 and M13mp18 single-stranded DNA. A mixture of pHY1603 and genomic DNA was loaded on the 6th to 9th lanes to precisely identify the positions of the SC and OC forms. Gel slices were prepared from the 6th to 9th lanes from left at the positions indicated by 1 to 8. The amounts of DNA loaded are as follows: genomic DNA, 880 ng; M13mp18 ssDNA, 200 ng; pHY1603, 172 ng (SC form for lane 3, linear form for lane 4, mixture of OC and SC forms for lane 5). **c** Quantitation of PCR products by microtip electrophoresis. Subpanel i, *attS* products. Subpanel ii, *gyrB* products. The numbers in the template row indicates the gel slice numbers in (b). PC, positive control (genomic DNA). NTC, no template control. The leftmost lane shows a size marker (bp) and internal standards (UM, LM) used for quantitation of each band. **d** The effect of restriction enzyme TfiI and single-strand-specific S1 nuclease on *attS* detection. The results of triplicate experiments are shown in parallel. No nuclease was added to the reactions in lanes labelled rCutSmart

To confirm the transposition route of SEs, genomic DNA of pSEA1-free *V. alfacsensis* strain BHY606 carrying a single copy of SE-6945 in the genome (see Method) was electrophoresed. Gel positions containing SE-6945 *attS* sequences were identified by PCR using outward-facing primers (Fig. 3a) to estimate the size and topology of *attS*-containing molecules. We prepared two molecules as analogs of a hypothetical circular transposition intermediate of SE-6945 (7,175 bp). One was a 7.2 kb circular plasmid pHY1603 carrying an *E. coli* chromosomal segment. The other was a single-stranded form of an M13 phage derivative M13mp18 (7.2 kb). These molecules were electrophoresed both separately and pooled with genomic DNA (Fig. 3b). According to qPCR experiments performed in a previous study [19], the *attL* × *attR* recombination products of SE-6945 and its copies are present at only 1 copy per 1000 chromosomal molecules in the cell population, thus the transposition intermediate is likely not detectable as a visible DNA band in an ethidium-bromide-stained gel (Fig. 3b, left lane). Fractions of genomic DNA were recovered from gel slices at the expected positions of the supercoiled form (SC in Fig. 3b; position 7), linear form (L; position 6), nicked circular form (OC; position 5), single-stranded circular form (position 2), other forms (position 3, 4) of the 7.2 kb plasmid DNA, and chromosomal DNA (position 1). Both the 1.0-kb segment containing SE-6945 *attS* and the 110 bp segment of *gyrB* in the chromosome were PCR-amplified. The amounts of PCR products were quantitated using the microtip electrophoresis system (Fig. 3c).

The 1 kb *attS* PCR products were obtained as expected when unfractionated genomic DNA was used as a template (PC in Fig. 3C), and were most efficiently obtained from gel slices from the position of the nicked circular form rather than the chromosome position, the single-stranded DNA position, or the supercoiled form position. The concentration of chromosomal DNA in the gel slices at the OC form position was less than in the single-stranded form and was approximately equivalent to that in the SC form according to the detection of *gyrB* (Fig. 3c-ii). This strongly suggests that the *attL ×* *attR* recombination products or their copies are mainly present as nicked and double-stranded circular DNA in cells. To validate this finding, genomic DNA mixed with MT13mp18 in a single-stranded form were treated with the single-strand-specific S1 nuclease or restriction enzymes TfiI. This process introduces double-stranded-breaks at several sites (Fig. 3a) in the targeted region of the hypothetical circular intermediate of SE-6945. PCR detection of *attS* was performed on the nuclease treated DNA (Fig. 3d). The *attS* product was almost undetectable for the genomic DNA treated with TfiI, but detectable in the genomic DNA treated with S1 nuclease at the same level as untreated DNA (rCutSmart in Fig. 3d). The activity of S1 nuclease was further confirmed by poor PCR detection of M13 gene III in M13pm18 single-stranded DNA added to the reaction mixture (Fig. 3d right).

The 1-kb *attS* PCR products amplified from the OC position were further cloned into the pGEM-T vector, then inserts of 23 clones were sequenced. All sequenced molecules contained the 6-bp sequence: 5′-TTTTTT-3′ next to the C end (Fig. 3a), but not 5′-TTTTCT-3′ next to the C′ end, confirming that strand-bias in *attL* × *attR* recombination is reflected in the circular SE copies as a characteristic 6-bp fingerprint.

Together, these results support the model that both the SE and host’s intracellular processes mainly produce SE copies in a double-stranded form (Fig. 2 left route), but not in a single-stranded form. Furthermore, the results indicate that the majority of circularized SE copies in the cells are nicked. Therefore, PCR products obtained using *attS*-targeting primer set (2 − 3 in Fig. 2) are regarded as PCR products of *attS* on the circular transposition intermediate of SEs.

### Core genes of SEs include four coding sequences

**Fig. 4:**
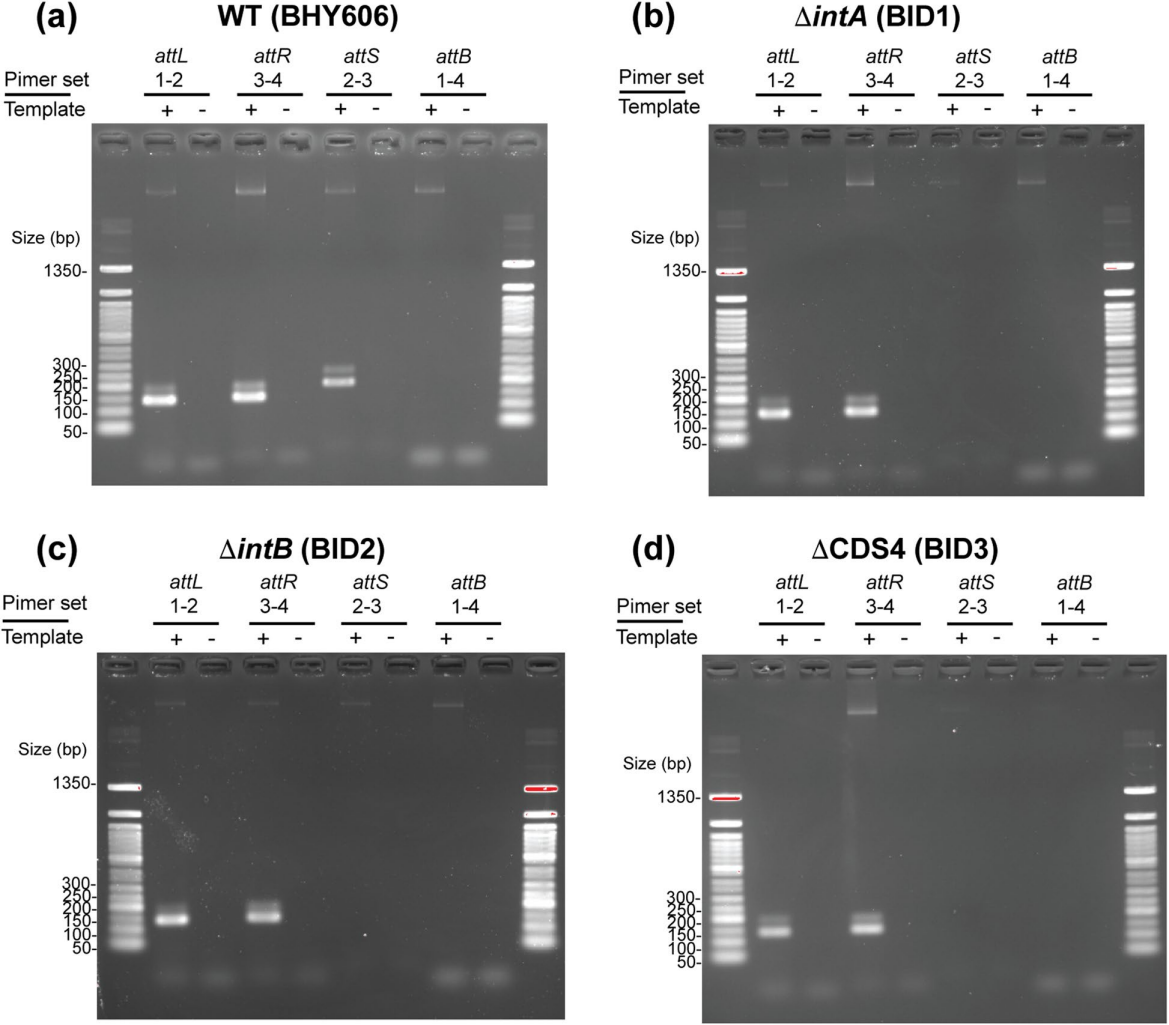
PCR detection of SE-6283 *attS* in single-gene knockout strains. **a** BHY606. **b** *intA*-knockout strain BID1. **c** *intB*-knockout strain BID2. **d** CDS4-knockout strain BID3. Primer numbers correspond to the numbers in Fig. 2a. PCR products were electrophoresed in 2.0% agarose gel. The ladder used was 50 bp DNA Ladder from New England Biolabs

Four genes have been previously identified to be conserved in several SE members in the genomes of multiple *Vibrio* species [19]. To determine whether these genes play roles in SE movements, we attempted to create single-gene deletion mutants of the four conserved SE genes (*intA*, CDS2, *intB*, CDS4) of SE-6283: the first discovered SE present in the chromosome of *V. alfacensis* 04Ya108. Next, the production of *attS* in mutant strains was investigated using PCR. A CDS2 deletion mutant could not be obtained despite repeated allelic exchange experiments. *attS* production was observed in BHY606 but not in the three single-gene knockout strains (Fig. 4). The complementation assay using the pBBR1MCS vector with the three knockout mutants restored the *attS* production for the CDS4 mutant but not for the *intA* and *intB* mutants (Additional file 1). Therefore, we conducted NGS (Next Generation Sequencing) on the mutants to investigate whether the genomes contain unexpected DNA rearrangements around the SE region. However, unexpected DNA rearrangement was not detected in the two *int* knockout mutants (data is available in Figshare). These suggest that an intact operonic structure of *intA-*CDS2*-intB* located at the motif C end as well as the presence or function of CDS4 independent of this operon are essential for the production of circular SE copies in vivo.

Based on these observations and a lack of specific motifs common to DNA-processing enzymes, the CDS4 product was concluded to be an auxiliary factor for recombination and named Srap (SE-associated recombination auxiliary protein). The protein structure prediction program Phyre2 [37] suggested that Srap has a motif similar to that of the TetR family transcriptional regulator from *Legionella pneumophila* (PDB ID: 3ON4) at the N-terminus but this finding has rather low confidence (51.4%). Jpred4 [38] revealed that both Srap and GinD of GInts possess a coiled-coil domain at the C terminus. The SE-6945 CDS2 product (WP_176703888.1) showed homology to Protein Data Bank (PDB) entries of several tyrosine recombinases with > 93% confidence (for example, PDB IDs: 5JJV and 1AIH) at the central part of the protein (positions 193–403) in Phyre2. However, the product of CDS2 was found to lack the common catalytic residues (RHRY motif) of tyrosine recombinases [20]. This is also true for GinC of GInts [23]. Thus, the CDS2 product might have a DNA-binding function but lack DNA-processing activity. Here, the CDS2 product has been named Tfp (tyrosine recombinase fold protein).

### SEs are mainly found in a subset of *Gammaproteobacteria*

To identify new SE members and their hosts, a synteny block of *tfp*-*srap* was searched for because of their uniqueness compared to other mobile DNA elements. The initial NCBI web server-based PSI-BLAST searches for non-redundant protein sequences (nr) did not identify the Tfp/Srap homologs in the genomes of microorganisms other than the *Gammaproteobacteria*. Therefore, we targeted the RefSeq genome database of *Gammaproteobacteria* (txid 1236) with assembly level “complete” (as of July 4, 2020), including sequence data of 15,358 replicons (chromosome or plasmid). The detailed screening method is described in the Methods section and Additional file 2.

Eleven rounds of joint analyses of PSI-BLAST searches with E-value cutoff 0.05 and genomic location searches for the coding sequences of PSI-BLAST hits (Tfp/Srap homologs) were performed and we identified 697 *tfp-srap* synteny blocks distributed in 561 replicons (comprising 3.6% of gammaproteobacterial replicons) spanning 48 genera. The number of unique ortholog sequences was 283 for Tfp (after filtering out the putatively truncated subjects with < 550 amino acids) and 308 for Srap. Tfp and Srap homologs were also searched for in the RefSeq protein database of the classes *Alphaproteobacteria* and *Betaproteobacteria*. However, no homologous sequences were identified. Furthermore, none of the products of *ginD* from 20 strains carrying GInt with identifiable termini listed by Bardaji et al*.* [23] were detected as PSI-BLAST hits of Srap. Therefore, some conserved amino-acid residues from SE homologs may not be fully conserved in GInt homologs. The genomic locations of the CDSs of Tfp/Srap orthologs are listed in Additional file 3.

**Fig. 5:**
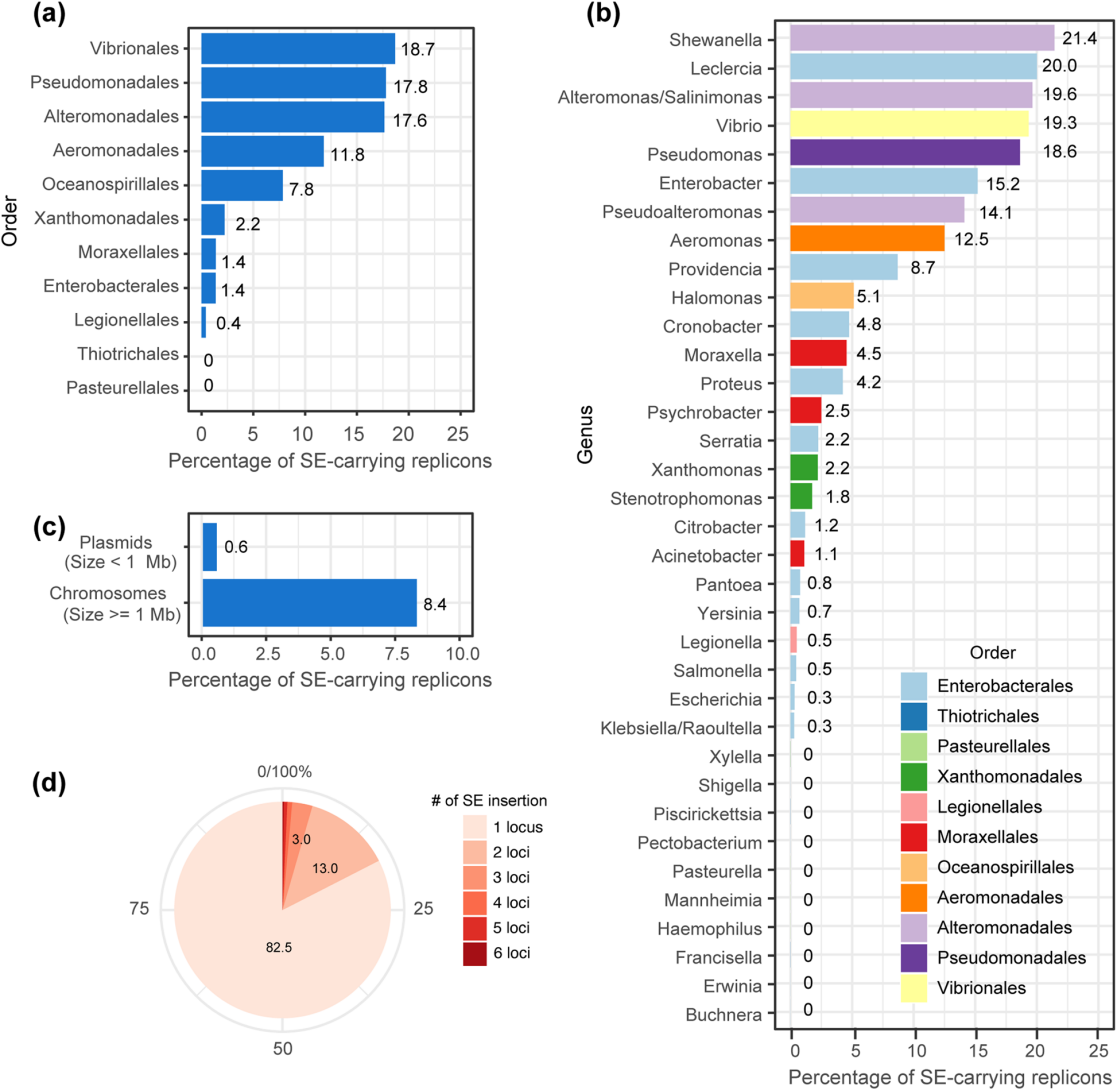
Hosts of the SEs. **a** Distribution of SEs in 10 orders that contain over 100 replicon entries in the database. The source data is in Additional file 4. **b** Distribution of SEs in 35 genera containing over 50 replicon entries. **c** Distribution of SEs in two types of replicons. One group with a small size (< 1 Mbp) was regarded as plasmid (*n* = 8527) whereas the other as a chromosome (*n* = 5613). The data originates from filtered genera that contain at least one SE. **d** Pie chart showing the distribution of insertion number of SEs per replicon. Insertion number patterns were classified into one insertion location (1 locus, a total of one SE) to six insertion locations (6 loci, a total of six SEs including both identical and distinct SEs)

To obtain further insights into the host range of SEs, the NCBI Taxonomic IDs linked to RefSeq genomes were retrieved, and the discovery rate of the SE-carrying replicons was calculated for each taxonomic group (Fig. 5a). At the order level, the discovery rate was the highest in *Vibrionales* (18.7% of replicons), followed by *Pseudomonadales* (17.8%), *Alteromonadales* (17.6%), and *Aeromonadales* (11.8%). SEs were not detected in the order *Pasteurelalles* and *Thiotrichales*. At the genus level, the discovery rate of SE-carrying replicons was the highest in *Shewanella* (21.4%), followed by *Lecleciera* (20.0%), *Alteromonas* (19.6%), *Vibrio* (19.3%), and *Pseudomonas* (18.6%). These genera are characterized as marine bacteria [39–44]. Although SEs were detected in *Enterobacteriales*, members of the genera *Erwinia* and *Buchnera*, both belonging to the family *Erwiniaceae*, were not detected as natural hosts of SEs (Fig. 5b).

SE-6283 was discovered on a conjugative plasmid pSEA1 of the *Vibrio alfacsensis* strain 04Ya108 which also had another copy of SE-6283 integrated into the chromosome [19]. Thus, SEs were initially hypothesized to be distributed at a similar frequency on the plasmids and chromosomes. However, on average in the *Gammaproteobacteria*, SEs were more frequently detected in chromosomes than in plasmids (8.4% versus 0.6%) (Fig. 5c). When an SE was present, the insertion number in one replicon was only one in 82.3% of the cases and two in 13.2%. Cases with more than two insertions were very rare (< 0.8%) (Fig. 5d), suggesting the presence of constraints in intragenomic SE amplification. This might be associated with deleterious homologous recombination between the SE copies or the target site preference of the SEs.

### Association between the SE gene product sequence and host taxonomy

**Fig. 6:**
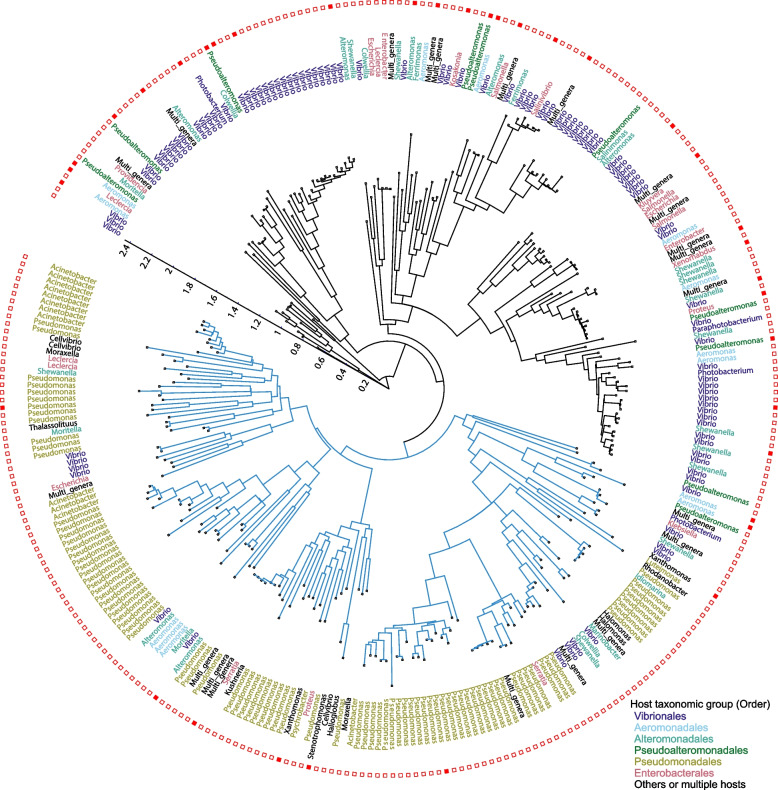
Phylogenetic tree of SEs. The tree was constructed based on the Srap alignment using IQ-tree2 NONREV model [45]. A consensus tree is shown. An SE clade detected in *Pseudomonadales* is indicated by blue lines. The color of the host genus denotes the host order. Leaves indicated by the red filled squares are SEs with identifiable termini illustrated in Figs. 1 and 7, or Additional file 6. The tree is annotated and visualized using iTOL [46]

A phylogenetic tree of SE based on the Srap ortholog sequence alignment is shown in Fig. 6. The frequent observation of *Pseudomonas* and *Vibrio* in the terminal nodes suggests the progressive diversification of the SEs in these two genera. Surprisingly, the genus *Pseudomonas* was detected as the hosts only in one large SE clade highlighted in the phylogenetic tree (blue lines in Fig. 6). A similar host separation was also observed in the SE tree based on the Tfp alignment (Additional file 5)*.* Therefore, amino-acid substitutions occurring in a common ancestor of specific SE clades seem to have expanded the host range to *Pseudomonadales.*

### Transmission of the SE members beyond taxonomic borders

**Fig. 7:**
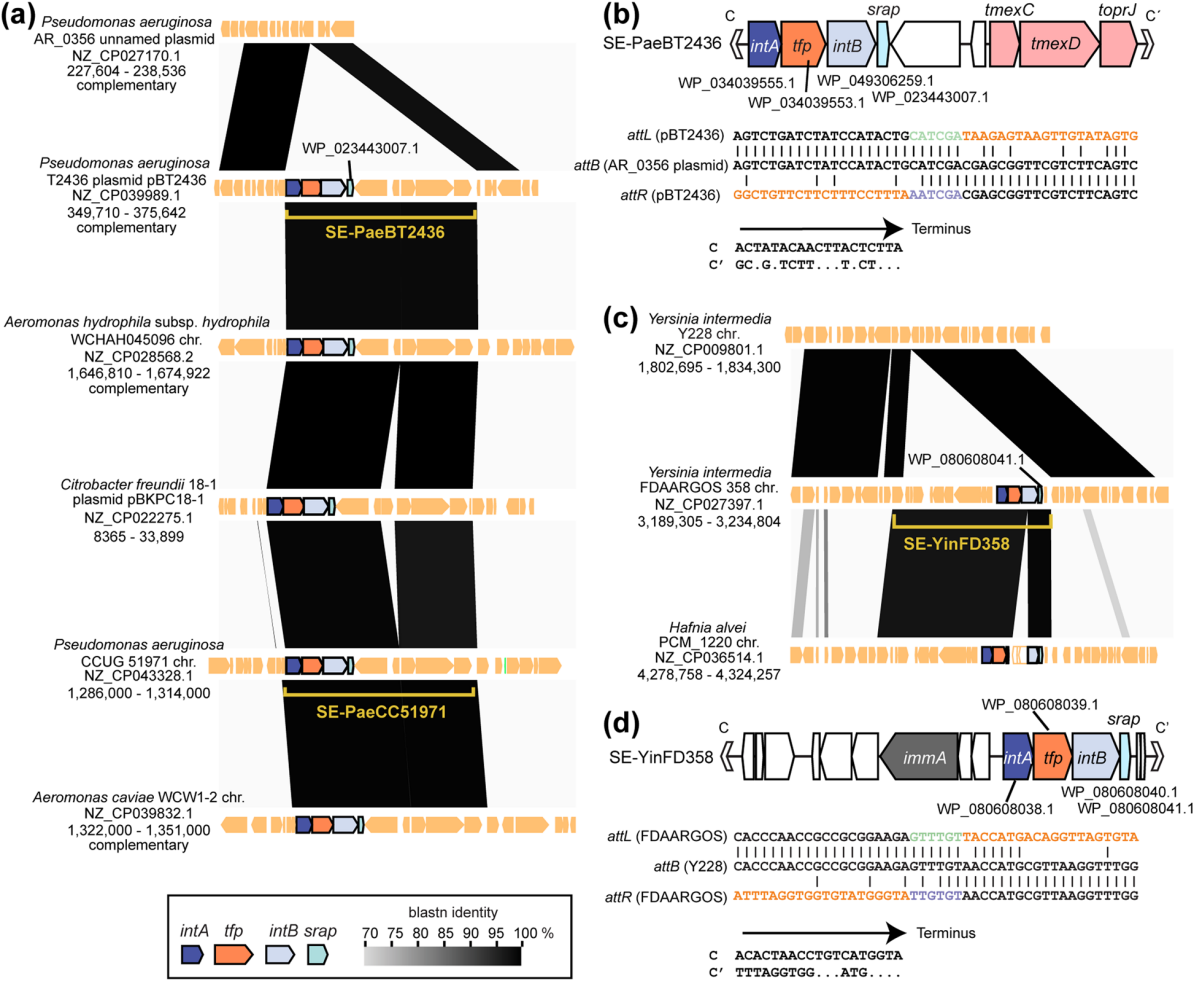
Transmission of SEs via transposition beyond the taxonomic borders of family. **a** Transposition of SE-PaeBT2436 and its related element SE-PaeCC51971. **b** Genetic organization of SE-PaeBT2436 and alignment of its terminal sequences with *attB*. **c** Transposition of SE-YinFD358. The *intB* homolog in the strain PCM_1220 is disrupted by an insertion. **d** Genetic organization of SE-YinFD358 and alignment of its terminal sequences with *attB*

Protein IDs associated with SEs are often linked to multiple species or genera, however, they are rarely found in multiple strains beyond the borders of families. Two SEs were detected in multiple families. One of them was named SE-PaeBT2436 (ID pair: WP_034039553.1 – WP_023443007.1, Fig. 5a and b). It carries a set of coding sequences of drug efflux RND transporter subunits and outer membrane protein (closest to TmexC3, TmexD3, ToprJ1 in the AMRFinderPlus database [47]), which collectively confer tetracycline and tigecycline resistance [48]. SE-PaeBT2436 and its close relative SE-PaeCC51971 (ID pair: WP_034066275.1 – WP_079388256.1) are found in the chromosomes or plasmids from the genera *Pseudomonas* (family *Pseudomonadaceae*), *Aeromonas* (*Aeromonadaceae*), and *Citrobacter* (*Enterobacteriaceae*), indicating SE transmission via multiple transposition events. Furthermore, SE-PaeBT2436-like SEs were embedded in conjugative plasmids were either embedded in conjugative plasmids or in one case, nested within an ICE highlighted in the previous AMR-associated genomics studies [48–51](Additional file 7). The other SE, SE-YinFD358 (ID pair: WP_080608039.1 – WP_080608041.1) from the genus *Yersinia* (*Yersiniaceae*) was also identified in the genus *Hafnia* (family *Hafniaceae*); *intB* was disrupted by insertion into the *Hafnia* genome (Fig. 7c and d). SE-YinFD358 carries an *immA* homolog having functional equivalence to the anti-repressor of ICE*Bs1* [52]. The SE-PaeBT2436 is located on a potentially conjugative plasmid in multiple strains, which reinforces the role of plasmids in the interfamilial transmission of SEs.

### Size, G + C content, and AMR genes of the new SE members

To define the basic genetic features of SEs, the termini of putative SEs inserted in the genomes were inferred by comparing the genome structure between the two genomes, one containing putative SE core genes and one without. Besides the two previously described SEs (SE-6945 and SE-6283) and two putative SEs (SE-YinFD358, SEs-PaeBT4236) described above, the termini of 33 new SE members were identified based on the alignment of *attL*, *attR*, and *attB* (Additional file 6). One SE from *Xanthomonas campestris* (SE-XcaCN17 in NZ_CP017307) was found to carry *srap* in an orientation opposite to that of the other three SE core genes. So far, SE-XcaCN17 is the only example possessing this atypical gene orientation. The median size of the 37 SEs was 15,688 bp, with a minimum length of 6,353 bp (SE-AmaTe101 carrying no cargo gene) and a maximum length of 58,529 bp (SE-PamAT11528) (Additional file 6 and Additional file 8). Most SE core genes identified in the chromosomes of genera *Pseudomonas* and *Acinetobacter* were embedded in a plasticity region, thus the exact SE termini could not be identified (examples are shown in panels (i) and (ii) in Additional file 6).

**Fig. 8:**
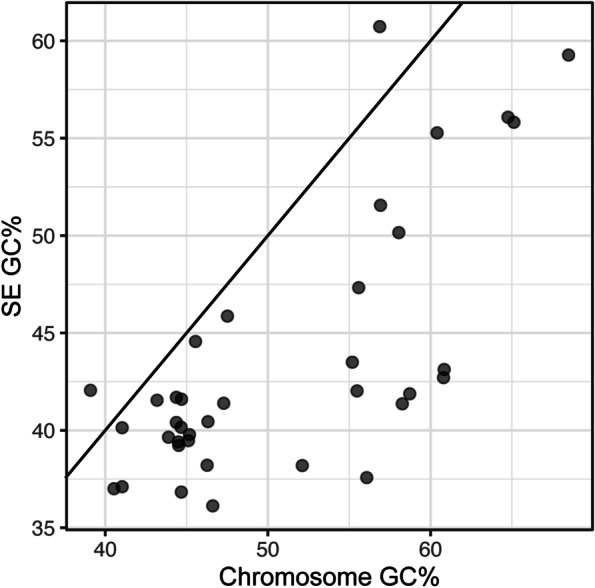
Association between the G + C percentages of SE and host species chromosome. The line in the plot is a linear model of Y = X

Previous base composition studies have repeatedly demonstrated a correlation between the G + C percentage of parasite (or symbiont) DNA, such as plasmids, phages, and ISs, and with the host G + C percentage [53–56]. The G + C percentages of the parasite DNA are generally lower than the host chromosome, possibly due to natural selection reducing overall fitness cost [56]. Further investigations focused on whether these rules of G + C content of parasite DNA also applied for SEs. G + C percentages of 37 SE regions were plotted against the G + C percentage of the paired chromosomes in Fig. 8. Similar to plasmids, the G + C percentage of these SEs was positively correlated with the G + C percentage of chromosomes (Pearson’s correlation coefficient *r* = 0.740), and the values were lower than the host chromosome in 94.6% of cases.

The antimicrobial resistance genes embedded within the 37 SEs were identified using AMRFinderPlus [47]. The coding sequence of the GMA family class A β-lactamase was detected in both SE-6945 (*bla* in Fig. 1a; locus_tag VYA_04440) and an SE (SE-Pda04Ya311) in the plasmid pAQU1 from *Photobacterium damselae* aquaculture sediment isolate. An SE in *Leclercia adecarboxylata* G426 chromosome (SE-LadG246) has the coding sequence of the MCR-9 family phosphoethanolamine-lipid A transferase (locus_tag FY047_06445), which likely confers colistin resistance. The SE-PaeBT2436 encodes the tetracycline/tigecycline resistance transporters, as described above. However, no AMR genes were detected in the remaining 33 SEs.

### Three newly identified SE members elicit strand-biased recombination activity

**Fig. 9:**
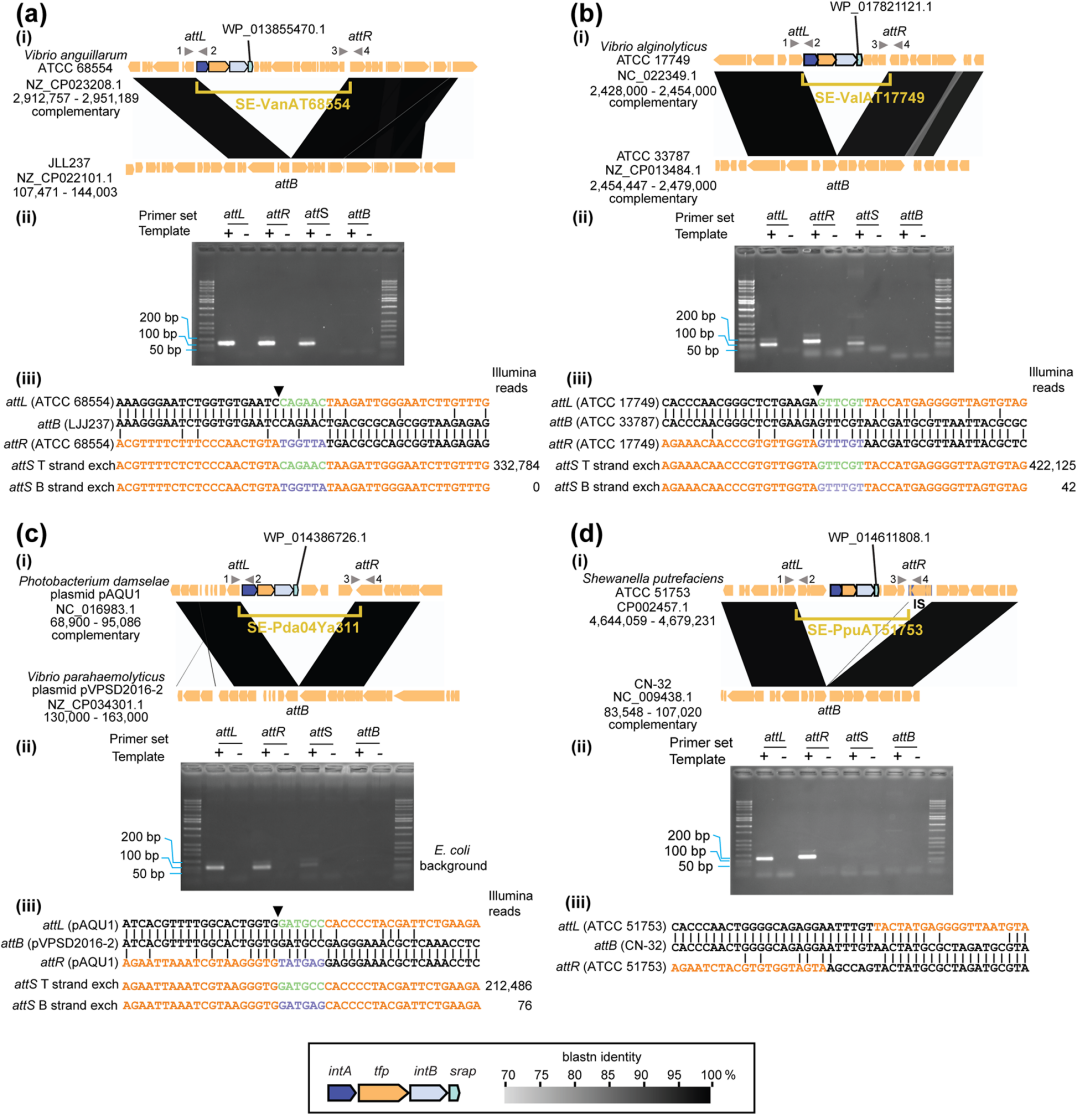
Strand-biased *attL* × *attR* recombination of putative SEs. **a** SE-VanAT68554 from *V. anguillarum.* (i) Comparison of the genome structures based on blastn. Primers used for *att* sites detection PCR were depicted by numbered triangles*.* (ii) Results of the *att* site detection PCR. The primer set used was 1–2 for *attL*, 3–4 for *attR*, 2–3 for *attS*, and 1–4 for *attB*. (iii) Alignment of *attL*, *attB*, and *attR*. Only the top strand is shown. SE region is shown in orange. The expected *attS* sequences (T strand exch, top strand exchange product; B strand exch, bottom strand exchange products) are shown below the alignment with their observed number of Illumina reads. The black triangle indicates the putative nicking site in *attL* × *attR* recombination. **b** SE-ValAT17749 from *V. alginolyticus*. **c** SE-Pda04Ya311 from the *Photobacterium* plasmid pAQU1. Genomic DNA extracted from *E. coli* W3110rif carrying pAQU1 was used as the template. **d** SE-PpuAT51753 from *S. putrefaciens*. Note that IS was inserted into the *attR* region. Takara Wide range DNA ladder (Takara Bio, Shiga, Japan) was used as ladder marker across panels

To investigate whether the bioinformatically screened SE members share features with SE-6945 and SE-6283 and possess the expected termini, PCR detection of *attS* was conducted for three American Type Culture Collection (ATCC) strains carrying only one copy of SE in the genome and one *E. coli* strain carrying plasmid pAQU1 obtained in our previous study [57] (Fig. 9 a–d). Here, four primers were designed per SE to obtain four primer sets amplifying *attL*, *attR*, *attS*, and *attB*, as shown in Fig. 2a. When SEs were active, we expected to detect *attS* but not *attB.* PCR products of *attL* and *attR* were also amplified to ensure primer functionality. *attS* generation was detected for the putative SEs in the three strains but not detected for one putative SE in *Shewanella putrefaciens* ATCC 51753. Empty *attB* was not detected in the three strains that produced *attS*.

To assess whether the three new active SE members also had strand bias in *attL* × *attR* recombination, sequence variations in the *attS* spacer region were analyzed by deep sequencing of *attS* PCR products. We assume that top strand exchange generates *attS* containing a 6-bp spacer originating upstream of the motif C terminus of the SE, whereas bottom strand exchange generates a 6 bp spacer originating from downstream of the motif C´ terminus. If the three putative elements were SE, their *attS* sequences should predominantly originate from top strand exchange products. A similar analysis was performed for *V. alfacsensis* BHY606 carrying a single copy of SE-6283 as a control. The results are summarized in Fig. 9 (iii). The detailed read content is shown in Additional file 9. The bottom strand change products were rarely detected for an SE from ATCC 17749 (SE-ValAT17749) (< 0.010% of total joints) and SE-Pda04Ya311 from the plasmid pAQU1 (0.035%) and were not observed at all (< 0.001%) for an SE from ATCC 68554 (SE-LanAT68554) and SE-6283. Top strand exchange products originating from the secondary nicking sites [18] were detected for SE-6283 at a 1.2% frequency of products from the primary nicking site. However, this secondary nicking site was not detected in the three new SE members through amplicon sequencing. Together, these results show that the unique movements of the two previously characterized SEs (SE-6283 and SE-6945) are also conserved in the three new SE members.

### SE and GInt are distinguishable groups

The evolutionary relationship between SEs and GInts was previously unknown. Since the use of SE proteins as PSI-BLAST queries did not detect a GInt synteny block, we hypothesized that GInt proteins and SE proteins respectively form a distinct ortholog cluster. To validate this, synteny blocks of *ginC- ginD* of GInts were searched for in the RefSeq dataset using GinC and GinD of GInt-DS1466 (Fig. 1a) as starting queried sequences for PSI-BLAST. Then, curated GInt proteins were pooled with SE proteins, and protein similarity networks based on BLAST E-values were constructed.

**Fig. 10:**
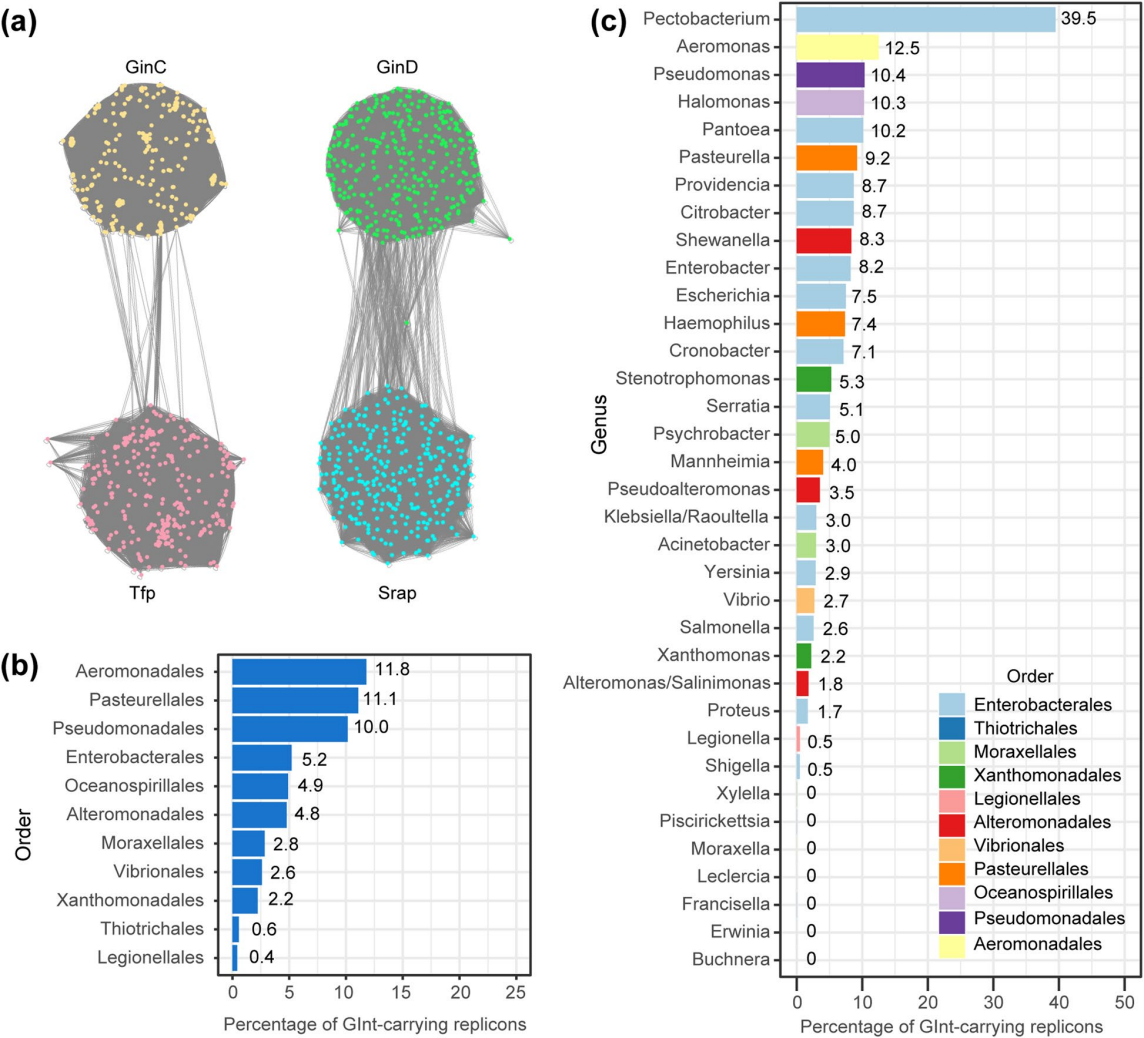
Similarity between GInt and SE proteins and hosts of the GInts. **a** Protein similarity networks of GinC/Tfp and GinD/Srap. Nodes are positioned using edge-weighted spring embedded layout in Cytoscape [58]. The edges represent BLASTP E-values. Node color indicates MCL cluster, and corresponds to GinC, Tfp, GinD, or Srap. **b** Distribution of GInts in 10 orders of *Gammaproteobacteria*. The source data is in Additional file 10. **c** Distribution of GInts in 35 genera

A total of 409 unique *ginC-ginD* synteny blocks were identified in 766 (5.0%) of gammaproteobacterial replicons spanning 52 genera (Additional file 10). The *ginC-ginD* synteny blocks were also identified in *Betaproteobacteria* (69 replicons comprising 2.5% of betaproteobacterial replicons), but not at all in *Alphaproteobacteria*. A total of 329 GinC proteins and 335 GinD proteins were retrieved from Gamma and Betaproteobacteria datasets. As expected, most GinC proteins (tyrosine recombinase fold protein lacking catalytic tyrosine) did not hit against Tfp proteins by BLASTP and vice versa (Fig. 10a). MCL clustering with inflation value (I) = 1.1 split GinC/Tfp network into a GinC cluster and a Tfp cluster. Similarly, MCL clustering with I = 1.1 split GinD/Srap network into a GinD cluster and a Srap cluster. Therefore, GInt and SE can be handled as different mobile DNA element groups based on their gene product sequences, and they are likely independently diversified groups.

To address the host range of GInts, GInt discovery rates in *Gammaproteobaeria* were determined and are summarized in Fig. 10b and c. GInts were most frequently detected in the orders *Aeromonadales* (11.8% of replicons), *Pasteurellales* (11.1%), *Pseudomonadales* (10.0%), followed by *Enterobacterales* (5.2%). At the genus level, GInts were most frequently detected in genus *Pectobacterium* (39.5%), followed by *Aeromonas* (12.5%), *Pseudomonas* (10.4%), *Halomonas* (10.3%), and *Pantoea* (10.2%). GInts were more prevalent than SEs in genus *Pectobacterium* (39.5% vs 0%), *Pantoea* (10.2% vs 0.8%), *Escherichia* (7.5% vs 0.3%), *Klebsiella/Raoultella* (3.0% vs 0.3%), and less prevalent in genus *Pseudomonas* (10.4% vs 18.6%), *Shewanella* (8.3% vs 21.4%), and *Vibrio* (2.7% vs 19.3%) (Figs. 10a and 5b). Therefore, besides sequence and gene orders, host range and host preference are clearly different between SEs and GInts.

## Discussion

**Fig. 11:**
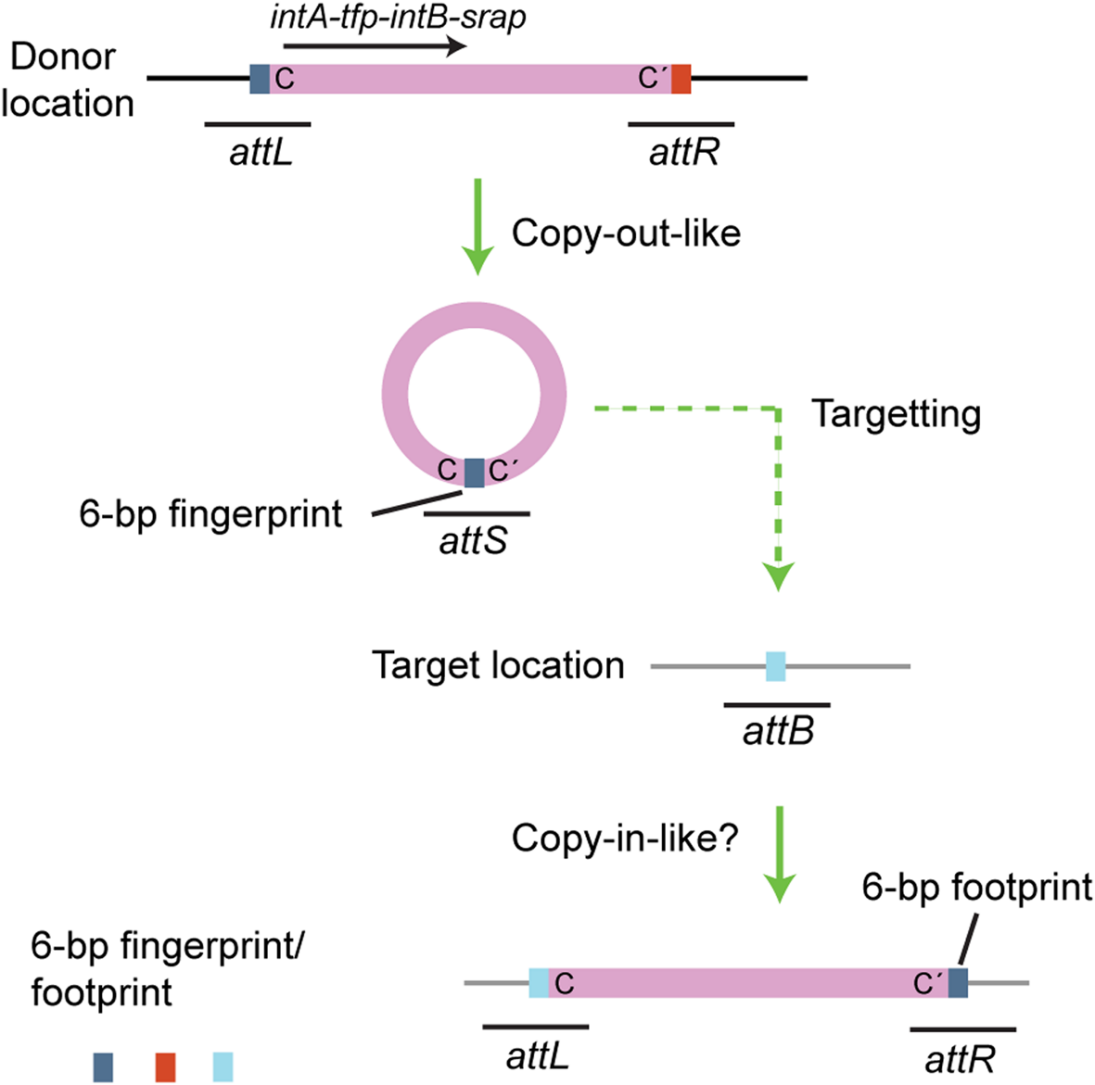
Feature of SE movement. SEs produce double-stranded DNAs containing the 6 bp of the left flank as transposition intermediates through a copy-out like route. After completion of integration, 6 bp in *attB* is placed at *attL* and the 6 bp fingerprint is placed at *attR*. Double-stranded DNA is shown as a single line

This study aimed to determine the host range of SEs which were rarely reported and overlooked as mobile DNA units in previous antimicrobial resistance-associated surveillance studies [48–51]. To date, five prokaryotic DNA transposon groups that encode tyrosine recombinases have been recognized: ICE/IMEs [9, 59], SEs [18, 19], GInts [23], RITs [24–26], and Tn*554* [30, 60] (Table 1). To the best of our knowledge, no transposition of RITs and GInts has been reproduced in the laboratory. The movements of the well-characterized ICE/IMEs all equal the ‘cut-out paste-in’ (excision/integration). Most ICE/IMEs encode a tyrosine recombinase and the recombination directionality factor Xis for strand nicking and exchange [16, 32–34]. SEs/GInts encode four conserved proteins involved in in vivo site-specific recombination, one of which (Tfp/GinC homolog) is unique to SEs/GInts. Observations on SE movement obtained in this study and previous studies are summarized as Fig. 11. New experimental evidence added in this study indicate the occurrence of a double-stranded circular form of an SE in genomic DNA. This supports the model that SEs transmit using a copy-out-like route. The circular form of SEs—the OC form containing only left flank of the donor location in *attS—*contrasts with an ICE circular form that is supercoiled and contains both left and right flanks of the donor location at the joint region [22, 61]. Integration of nicked circular SE copies (*attS* × *attB* recombination) might also occur through recombination of one specific strand of both *attB* and *attS*, since the 6-bp fingerprint in *attS* was placed in the newly formed *attR* in all three SE integration events observed in our previous studies: the SE-6283 insertion into *bcp* [18] and the SE-6945 insertion into *insJ* of IS*3* and *yjjNt* in the *E. coli* chromosome [19]. Therefore, in the case of SEs, ‘copy-out copy-in’ is a more likely transposition mode than ‘copy-out paste-in’. The characteristic feature of copying 6-bp from the left side of the donor location into the right side of the new insertion location leaving a 6-bp footprint (Fig. 11) is also observed for transpositions of Tn*554*-related elements [27, 30]. Tn*554* transposition depends on TnpC [28] similar to the Srap-dependence of *attL* × *attR* recombination of SE-6283. Therefore, the transposition mechanism of Tn*554* might be similar to SEs although a Tfp counterpart is lacking in Tn*554*. Evolutionary relationship among SEs, GInts, RITs, and Tn*554* are still vague and require further investigation regarding differentiation of circularization and integration systems.

**Table 1:** Features of five prokaryotic DNA transposon groups encoding tyrosine recombinases

	ICEs/IMEs	SEs	GInts^c^	RITs^d^	Tn*554*-related elements^e^
Hosts	Archaea Bacteria	Gammaproteobacteria	Gammaproteobacteria Betaproteobacteria	Bacteria	*Staphylococcus* *Enterococcus*
Proteins involved in recombination	Int, Xis	IntA, Tfp, IntB, Srap	GinA, GinB, GinC, GinD	RitA, RitB, RitC	TnpA, TnpB, TnpC
Target site specificity^a^	+	+	+	NT	+
Empty site^b^	+	−	±	NT	NT
Movement	Cut-out paste-in (excision integration)	Copy-out copy-in?	NT	NT	NT

^a^+ : The presence of target site specificity. NT: not formally tested by experiments

^b^+ : *attL* × *attR* recombination produces empty site. -: *attL* × *attR* recombination does not produce empty site. ± : some members do not produce empty site

^c^Bardaji et al*.* [23] mentioned the presence of GInts in taxa other than *Proteobacteria*. However, the discovery rate is unknown

^d^The RIT elements (RITs) comprise three coding regions encoding tyrosine recombinase and 34 bp terminal inverted repeats [24–26]. Three recombinases of archetype RIT element in *Cupriavidus metallidurans* CH34 (NC_007973.1; locus_tags Rmet_1271, Rmet_1272, Rmet_1273) are 315 to 410 aa long in size, and all possess catalytic tyrosine

^e^The host range of Tn*554-*related elements and whether Tn*554-*related elements generate empty sites have not formally been investigated. TnpC is the Srap/GinD counterpart according to structure prediction

As initially speculated, the SE members were identified at a high frequency (18%–21%) only in the several orders of the free-living *Gammaproteobacteria,* even though attempts were made to detect the distant homologs using PSI-BLAST. The SE discovery rate has been represented per replicon in Fig. 5. The SE discovery rate per strain is likely to be highest in the genus *Vibrio* as it usually carries two chromosomes. The four major host orders (*Vibrionales*, *Pseudomonadales*, *Alteromonadales*, and *Aeromonadales*) are all associated with marine environments [39, 41–44]. Thus, a common ancestor of SEs might have emerged in the *Gammaproteobacteria* living in marine environments.

This narrow host range of SEs might be due to the complexity of the strand exchange processes involving four proteins to achieve the copy-out-like movement. Similar to SEs, so far the only reported hosts of Tn*554*-related elements with a three-protein component system are in genera *Staphylococcus* and *Enterococcus* [29, 30] (Table 1). Amplicon sequencing revealed that the strand bias in *attL* × *attR* recombination of SEs is very strict (Additional file 9). Since transposition intermediates of SEs are double-stranded circular DNA (Fig. 3), the DNA–protein crosslink repair [62]-equivalent step should also be involved in the removal of the covalently linked integrase to generate 3′-OH as a replication priming site (Fig. 2). However, a tyrosyl-DNA phosphodiesterase [63] has not yet been identified in prokaryotes. Notably, the members of the specific SE clades (Fig. 5) were not detected in the *Pseudomonadales*. Therefore, interactions with specific host factors may also be involved in dissemination of SEs.

This study revealed that SEs and GInts are distinguishable by their sequences, and that GInts are distributed in a wider taxonomic range than SEs. Other than *Pectobacterium,* GInts appear to have no host preference, while SEs have a demonstrated preference to marine bacteria. Members of the genus *Pectobacterium* are known as plant pathogens [64]. GInt-*Pectobacterium* association may be due to the cargo genes carried by GInts as some GInts members carry pathogenicity island Pht-PAI [23]. Regarding size, SEs with identifiable termini (without GInt members) were a median length of 15.7 kb (*n* = 37) and were much smaller than the GInt median size (34.9 kb, *n* = 20 with identifiable termini). Therefore, the GInts have acquired many genes during their long-term association with the hosts. The SE core genes in the genera *Pseudomonas* and *Acinetobacter* are frequently embedded within a chromosomal plasticity region and not in the core region. Therefore, their termini could not be identified by simple genome comparison in this study. Most SEs in *Pseudomonadales* may hitchhike with other mobile genetic elements, such as ICE/IMEs, which have a mobilization capacity, like the SE embedded in an ICE in the *Proteus mirabilis* chromosome [51] (Additional file 7).

SE-PaeBT2436, which carries the *mexCD*-*oprJ* homolog (*tmexCD-toprJ*), is important as a carrier of antimicrobial resistance genes because of its pan-Asian transmission in clinical environments, as evident in previous antimicrobial-resistant bacteria surveillance studies [48–51]. This study is the first to clarify the mobility unit of SE-PaeBT2436 and provide mechanistic insights into DNA rearrangements around the *tmexCD*-*toprJ* cluster. Furthermore, the SE-LadG426 from the opportunistic pathogen *Leclercia adecarboxylata* strain G426 (blood isolate from China; NZ_CP043398.1) carried the *mcr-9* colistin-resistance gene (locus tag FY047_06445, protein id: WP_150870284.1). These SEs may be important for future epidemiological studies.

The reasons for the inability to knockout the *tfp* gene (CDS2) remain unknown. Altered expression levels of *intA* and *intB* due to *tfp* deletion might have a deleterious effect on the *Vibrio* host. Similarly, no restoration of *attL* × *attR* recombination in the *intA* or *intB* complementation strains was difficult to explain. It is reported that IS*903* does not move efficiently when the transposase gene is provided *in trans* to the terminal inverted repeats [65]. Furthermore, translational coupling is suggested from the overlap between coding region and S.D. sequence (*intA*-*tfp*, *tfp-intB, intB-srap*) of the SE genes. Therefore, ectopic gene expression from a plasmid vector might not be able to establish interactions among SE proteins at the right stoichiometry on *attL* and *attR*. These two findings suggest that the intact operonic structure of *attL* (promoter region)-*intA*-*tfp*-*intB* is essential for SE movement in vivo. Further biochemical studies are needed to address the specific roles of each SE protein in the strand nicking and exchange processes.

A limitation of this study lies in the PSI-BLAST approach, which might not detect some distant members of SEs. Other approaches might allow more divergent clades of SEs to be detected. Nevertheless, this study has detected very distant SE members (see alignments used in phylogenetic analysis), including members with unique genetic organization, such as SE-AmaTe101 and SE-XcaCN17. Furthermore, this study provides quantitative information on the SE/GInt discovery rate per taxon. This information might serve as the foundation for future mobile DNA-host coevolution studies.

## Conclusions

Genomic DNA fractionation experiments suggested transposition intermediates of SEs to be double-stranded and nicked circular form. Molecular genetics experiments have revealed SE core genes to be essential for *attL* × *attR* recombination. Synteny block searches in the RefSeq complete genome sequence dataset revealed the SEs to be mainly distributed in several orders of *Gammaproteobacteria*. The three newly identified SE members showed strand-biased *attL* × *attR* recombination activities.

## Methods

### Bacterial strains, plasmids, and culture media

The strains and plasmids used in these experiments have been summarized in Table 2. *E. coli* was cultured in LB broth Lennox (Nacalai Tesque, Kyoto, Japan), whereas *Vibrio* and *Shewanella* strains were cultured in the solid medium comprising the BD Difco™ Marine Broth 2216 (Becton, Dickinson, and Company) supplemented with 1.5% agar. The *V. alfacsensis* strains were cultured in the LB broth supplemented with up to 2% NaCl (LB-M) or BBL™ Brain Heart Infusion (BHI) broth supplemented with up to 2% NaCl.

**Table 2:** Strains and plasmids used

Strains or plasmids	Genotype and relevant characteristics ^*a*^	Reference or source
*E. coli*		
MG1655	F^−^, λ^−^, *rph-1*	Type strain
HIT-JM109	*endA1*, *recA1*, *gyrA*96, *thi, hsdR*17 (r_k_^–^, m_k_^+^), *relA*1, *supE*44, Δ(*lac-proAB*), [F´ *traD36*, *proAB*, *laqI*^q^ZΔM15]	Real Biotech Corp., Taipei, Taiwan
DH5α	F^−^, λ^−^, *recA*1, *endA*1, *relA*1,*gyrA*96, *deoR*, *supE*44, *thi*-1, *hsdR*17(r_k_^–^, m_k_^+^), Φ80d*lacZ* ΔM15, Δ(*lacZYA-argF*)U169	Nippon Gene Co.Ltd
W3110Rif	λ^*−*^*, IN(rrnD-rrnE)1, rph-1*; spontaneous rifampicin-resistance mutant of W3110, Rif^r^	[66]
EC100pir +	F^−^*mcrA* Δ(*mrr-hsdRMS-mcrBC*) φ80dlacZΔM15 *ΔlacX*74 *recA*1 *endA*1 *araD*139 Δ(*ara*, *leu*)7697 *galU galK* λ^−^*rpsL nupG pir* + (DHFR); Sm^r^	Lucigen, Middleton, WI, USA
β3914	F^−^ RP4-2-Tc::Mu Δ*dap*A::(*erm-pir*) *gyrA462* *zei-298*::Tn*10*; Tc^r^, Km^r^, Em^r^	[67]
*Vibrio alfacsensis*		
04Ya108	Aquaculture isolate, carrying SE-6283 and SE-6945 in both chromosome 1 and plasmid pSEA1; Tc^r^, Ap^r^, Cm^r^, Erm^r^	[19]
BHY606	pSEA1-free derivative of 04Ya108; Ap^r^	This study
BID1	BHY606Δ*intA*_SE-6283_; Ap^r^	This study
BID2	BHY606Δ*intB*_SE-6283_; Ap^r^	This study
BID3	BHY606Δ*srap*_SE-6283_; Ap^r^	This study
*Vibrio alginolyticus*		
ATCC 17749	Spoiled horse mackerel isolate, carrying SE-ValAT17749	ATCC
*Vibrio anguillarum*		
ATCC 68554	Unknown isolate source, carrying SE-LanAT68554	ATCC
*Shewanella putrefaciens*		
ATCC 51753	Oil pipeline isolate, carrying SE-SpuAT1753 (inactive SE)	ATCC
Plasmids		
pAQU1	Mob_H_ family multi-drug resistance plasmid derived from *Photobacterium damselae* subsp. *damselae* strain 04Ya311, SE-Pda04Ya311; Cm^r^, Erm^r^, Tc^r^, Ap^r^	[66]
pBBR1MCS	Broad host range cloning vector; Cm^r^	[68]
pBBR-*intA*	pBBR1MCS carrying *intA*_SE-6283_; Cm^r^	This study
pBBR-*intB*	pBBR1MCS carrying *intB*_SE-6283_; Cm^r^	This study
pBBR-*srap*	pBBR1MCS carrying *srap*_SE-6283_; Cm^r^	This study
pGEM-T	Cloning vector for TA cloning	Promega, Middleton, WI USA
pGEM-*attS* (23 clones)	pGEM-T derivative carrying 1.0 kb segment derived from SE-6945 circular form; Ap^r^	This study
pHY1603	pSTV28 derivative carrying 4.2 kb *E. coli* chromosomal segment	This study
pIDO1	pSW7848 carrying 0.9 kb upstream and downstream of *intA*_SE-6283_; Cm^r^	This study
pIDO2	pSW7848 carrying 0.9 kb upstream and downstream of *tfp*_SE-6283_; Cm^r^	This study
pIDO3	pSW7848 carrying 0.9 kb upstream and downstream of *intB*_SE-6283_; Cm^r^	This study
pIDO4	pSW7848 carrying 0.9 kb upstream and downstream of *srap*_SE-6283_; Cm^r^	This study
pSTV28	Cloning vector; p15A *oriV*; Cm^r^	Takara Bio, Inc
pSW7848	Allele exchange vector for *Vibrio*; R6K replicon, Cm^r^	[69]

^a^Abbreviations used for the antimicrobial resistance phenotypes (^r^) are as follows: *Cm* Chloramphenicol, *Erm* Erythromycin, *Tc* Tetracycline, *Ap* Ampicillin, *Rif* Rifampicin, *Sm* Streptomycin

The tetracycline-susceptible pSEA1-free strain BHY606 was constructed by two rounds of batch culture transfer of the strain 04Ya108 in fresh BHI 2% NaCl broth, plating the culture on marine broth agar plates, and the subsequent screening for tetracycline-susceptible colonies.

The allele exchange plasmids used for single-gene knockout of BHY606: pIDO1 (*intA* knockout), pIDO2 (CDS2/*tfp* knockout), pIDO3 (*intB* knockout), and pIDO4 were constructed as follows. First, the regions approximately 900 bp upstream and downstream from the target locus were PCR-amplified using the KOD plus neo high-fidelity DNA polymerase (Toyobo Co., Ltd.) and primers listed in Additional file 11. PCR products were purified using E-Gel CloneWell (Invitrogen, Waltham, MA, USA) and recombined with XbaI-HindIII-digested linearized pSW7848 using the NEBuilder HiFi DNA Assembly Master Mix (New England Biolabs, Ipswich, MA, USA). The reaction mixture was introduced into EC100pir + competent cells, and the Cm resistant clones were selected in the presence of 30 mM glucose. The resulting pSW7848 derivatives with the expected inserts were named pIDO1 (used for SE-6283 *intA* knockout), pIDO2 (SE-6283 CDS2/*tfp* knockout), pIDO3 (SE-6283 *intB* knockout), and pIDO4 (SE-6283 CDS4/*srap* knockout).

Plasmids for the complementation test pBBR-*intA*, pBBR-*intB*, and pBBR-*srap* were constructed by combining PCR-amplified SE-6283 genes into XbaI-HindIII-digested pBBR1MCS using NEBuilder (New England Biolabs). SE-6945 circular transposition intermediate analog pHY1603 was constructed by combining PCR-amplified 4.2 kb *E. coli* MG1655 chromosomal segment and PCR-amplified 3.0 kb vector pSTV28 (Takara Bio, Shiga, Japan), using NEBuilder (New England Biolabs). Primers used are listed in in Additional file 11.

### Molecular genetics experiments

The allele exchange plasmids pIDO1, pIDO2, pIDO3, and pIDO4 were introduced into strain β3914 by electroporation, and transformants were mated with BHY606 on an LB-M agar plate in the presence of 30 mM glucose and 300 µM diaminopimeric acid (DAP) at 30 °C overnight. The mating mixture was serially diluted in 1 × PBS and then plated on LB-M containing 30 mM glucose and 25 µg/mL chloramphenicol (Cm). After incubation at 30 °C overnight, a few colonies were picked and streaked on agar plates. About 20 to 300 colonies were replicated on the LB-M agar plates with Cm and the others on the plates without Cm. The Cm-sensitive clones were further screened for the absence of the original locus (full-length coding region) and the presence of the gene-depleted region by colony PCR. KOD One*®* (Toyobo Co., Ltd.) was used for confirming gene deletion by colony PCR.

For complementation test pBBR-*intA*, pBBR-*intB*, and pBBR-*srap* were introduced into strain β3914 by electroporation. Then, the transformants were mated with knockout mutants. *V. alfacensis* mutant strains carrying pBBR1 derivatives were further screened on LB-M agar plates containing Cm.

### Genomic DNA fractionation and microchip electrophoresis

Genomic DNA of BHY606 was prepared using Qiagen Genomic-tips 100/G column, Genomic DNA Buffer Set (Qiagen, Hilden, Germany), and Qiagen Proteinase K, following the manufacturer’s protocol. 880 ng genomic DNA was loaded onto 0.8% agarose gel prepared in 0.5 × Tris–Acetate-EDTA (TAE) buffer. Electrophoresis was performed for 90 min at 135 V. Gel slices were made from the ethidium bromine-stained gel using FastGene™ gel cutter (Nippon Genetics Co. Ltd.). DNA was purified from the gel slices using NucleoSpin® Gel and PCR clean-up kit (Takara Bio), and was eluted in 40 μL UltraPure™ DNase⁄RNase-free distilled water (Invitrogen).

PCR detection of the *attS, gyrB* in gel extracted DNA, and M13 gene III was performed using the One*Taq®* Quick-Load 2X Master Mix with Standard Buffer (New England Biolabs). PCR reaction mixture was prepared in a 25 μL reaction volume. Thermal cycler condition used for the detection of *attS* for this assay was 95 °C 5 min, 35 cycles of 95 °C 15 s, 54 °C 15 s, and 72 °C 20 s, 72 °C 1 min. The conditions used for *gyrB* and gene III were identical to the conditions above except that the number of amplification cycles was set to 20. PCR products were diluted twofold (*attS, gyrB*), or threefold in TE (gene III), then subjected to microchip electrophoresis with Invitrogen™ SYBR™ Gold Nucleic Acid Gel Stain (Thermo Scientific) in Shimadzu MCE-202 MultiNA (Shimadzu, Kyoto, Japan). DNA-1000 reagent kit (Shimadzu) and 100 bp DNA ladder (Takara Bio) were used for the microchip electrophoresis. PCR product quantity was estimated using the MultiNA viewer software (Shimadzu). The 1-kb *attS* PCR products were cloned using pGEM-T vector systems (Promega) in *E. coli* JM109.

To address the effect of nuclease on the detection of *attS*, a mixture of BHY606 genomic DNA (500 ng) and M13mp18 single-stranded DNA (Takara Bio) (500 ng) was treated in a 20 μL reaction with TfiI (New England Biolabs) at 65 °C for 30 min or with S1 nuclease (Takara Bio) at 37 °C for 30 min. The reaction was stopped by addition of 20 μL of phenol/chloroform/isoamyl alcohol followed by vortexing. Treated DNA was purified by ethanol precipitation and resuspended in 40 μL distilled water. 1 μL was added to the PCR reaction as template for the detection of *attS* and M13 gene III.

### PCR detection of the *att* sites

PCR detection of the four *att* sites of SE-6283 (Fig. 4) and four putative SEs (Fig. 9) was performed using the One*Taq®* (New England Biolabs). Genomic DNA was prepared using the GenElute™ Bacterial Genomic DNA Kits (Sigma-Aldrich, St. Louis, MO, USA) (Fig. 9) or Qiagen Genomic-tips 100/G columns, and Genomic DNA Buffer Set (Qiagen, Hilden, Germany) (Fig. 4 and Additional file 1). All *att* site detection PCRs were performed in a 25 µL reaction volume containing 1 µL of genomic DNA normalized to 15 ng/µL, and 0.6 µM primers. Dimethyl sulfoxide was added to 0.03 µg/µL, specifically, to the *attR* amplification of BHY606. Thermal cycler condition used was 95 °C 5 min, 30 cycles of 95 °C 15 s, 54 °C 15 s, and 72 °C 20 s, 72 °C 1 min regardless of the targets.

### Resequencing and amplicon sequencing

To confirm the absence of unexpected mutations around the deleted gene, Illumina sequencing was performed for all three single-gene knockout mutants (BID1, BID2, and BID3) and the parent strain BHY606. Qiagen Genomic-tips 100/G columns and Genomic DNA Buffer Set were used to extract the genomic DNA for NGS. The library was prepared using a TruSeq DNA PCR-free kit (Illumina, Inc., San Diego, CA, USA) and was sequenced on the NovaSeq 6000 platform at NovogeneAIT Genomics (Singapore). The raw reads were trimmed using fastp [70] and assembled using the Unicycler [71]. To investigate the sequence heterogeneity in the joint region (*attS*) of C and C′ on the circular SEs, PCR products of *attS* were obtained by PCR amplification of total DNA with KOD plus neo polymerase using the primers listed in Additional file 10. Amplicons were then purified, indexed for multiplex sequencing, and sequenced on the MiSeq platform (Illumina, Inc.) at Fasmac Co., Ltd. (Atsugi, Kanagawa, Japan) to give 236,270 to 488,664 paired reads per amplicon. The raw reads were trimmed, merged using fastp [70], and then filtered to select the reads containing the correct primer sequence using the seqkit [72]. The number of unique merged reads was counted using the fastp-uniq function of the fastq-tools [73]. The commands used in NGS data analysis are described in the README file available in Figshare [74].

### Dataset

The faa and gff files of the NCBI RefSeq genome entries of *Gammaproteobacteria* (taxid:1236, 6596 genomes and 15,358 sequence regions available on July 7, 2020), *Alphaproteobacteria* (taxid:28,211, 1221 genomes and 3463 sequence regions available on Sept 5, 2020), and *Betaproteobacteria* (taxid:28,216, 1790 genomes and 2774 sequence regions available on Sept 5, 2020) were downloaded from the NCBI server via the NCBI Taxonomy site (https://www.ncbi.nlm.nih.gov/taxonomy). The contents in the faa files were deduplicated using the SeqKit [72] and used as the BLAST 2.9.0 + protein database.

### Survey of the SE core genes in the RefSeq complete genome sequence database

The method for detecting the *tfp* (CDS2)–*srap* (CDS4) synteny block is illustrated in Additional file 2. The distant homologs of Tfp and Srap were searched based on the PSI-BLAST [75] using an E-value cut-off of 0.05. The length (*L*) of the DNA segment starting from the coding sequence of the Tfp-hit and the coding sequence of the Srap-hit was calculated based on the coordinate information in the gff files. PSI-BLAST hit-containing RefSeq genomes with *L* shorter than 6500 bp and longer than 1200 bp were identified using the functions of R 4.0.3 [76] and R package rtracklayer [77]. The amino-acid sequences of Srap and Tfp homologs originating from the synteny blocks were retrieved. The phylogenetic distance between Srap homologs was calculated using the R package phangorn [78]. The most distant Srap homolog from the query (and its paired Tfp homolog) was used as a new query in the next iteration of PSI-BLAST. This cycle was repeated until the PSI-BLAST search converged or the most distant hit subject became the PSI-BLAST query of the previous round of search. Henceforth, eight rounds of PSI-BLAST searches starting from SE-6283 queries and three rounds of searches starting from SE-6945 queries were conducted. The Tfp-hits were found to contain products from pseudogenes and typical tyrosine recombinases with the RHRY motif due to partial similarity. Those listed as unwanted hit subjects were removed from the final list of synteny blocks in the gff format. The commands and R scripts used are available in Figshare [74].

The same approach was employed to search for *ginC-ginD* synteny blocks of GInts. GinC (WP_014595881.1) and GinD (WP_043942113.1) of GInt-PstDS4166 (Fig. 1a) were used as starting queries of PSI-BLAST with E-value cut-off of 0.05. The filtering for the length between GinC hit and GinD hit was set to shorter than 3000 bp and longer than 1200 bp. Three rounds of searches were conducted for the GInts.

### Search for the SE termini

Over 40 Srap protein IDs were randomly chosen among 308 PSI-BLAST hits, and the linked genomic locations in the RefSeq genome files were identified manually. Their sequences were compared with the equivalent locations of the SE-free genome of bacterial species using the blastn function implemented using the GenomeMatcher software [79]. The left and right borders (*attL* and *attR*) between the putative SE (or SE-containing genomic island) and a chromosome/plasmid backbone were identified using the dot plot function of the software. Then, putative *attL*, *attR*, and SE insertion sites in the SE-free genome (*attB*) were aligned. The genomic inserts were regarded as SE if (i) the split *attB* sequences were retained in the putative *attL* and *attR,* (ii) the joint region in putative *attL* × *attR* recombination products (copied to *attS*) could form imperfect inverted repeats, and (iii) there was a 6 bp spacer between the repeats in *attS*.

### Phylogenetic analysis

The phylogenetic tree for SEs was inferred based on the Tfp or Srap sequences. The product sequences originating from the *tfp-*(*intB*)*-srap* synteny block were retrieved, filtered based on protein length, and aligned using MAFFT (v7.407) with the L-INS-I option [80]. The phylogenetic tree for alignments was inferred by the maximum likelihood method using IQ-tree (v2.1.3) with the NONREV model and 1000 bootstrapping options, followed by a root-test [45]. The tree files were visualized and annotated using iTOL [46].

### Protein similarity network

Tfp (or Srap) and GinC (or GinD) proteins, originating from SE and GInt synteny blocks were pooled, then all-to-all BLASTP was conducted with option of '-evalue 0.05 -outfmt 6'. BLAST results were reformatted for network visualization in Cytoscape v3.9.1 [58] and MCL clustering [81]. MCL v14-137 was used to split protein similarity network into ortholog clusters. MCL commands used are the followings: “mcxload -abc Tfp_GinC_abc.txt –stream-mirror –stream-neg-log10 -stream-tf 'ceil(200)' -o Tfp_GinC.mci -write-tab Tfp_GinC.tab” and “mcl Tfp_GinC.mci -I 1.1 -use-tab Tfp_GinC.tab”.

### Host taxonomy information

NCBI Taxonomy IDs linked to the RefSeq genomes were retrieved from the gff files (“taxon” in the gff file), and their linked order, family, and genus were obtained using the TaxonKit software [82]. Information on the RefSeq ID, taxonomy, and presence of SE is summarized in Additional file 4.

## Supplementary Information

**Additional file 1. **Results of the gene complementation test. Primer numbers correspond to the numbers in Fig. 2. (i) Strain BID1 harboring pBBR1MCS, (ii) strain BID1 harboring pBBR-intA, (iii) strain BID2 harboring pBBR1MCS, (iv) strain BID2 harboring pBBR-intB, (v) strain BID3 harboring pBBR1MCS, and (vi) strain BID3 harboring pBBR-srap. Electrophoresis was performed using a 2% agarose gel. The faint band in the attR PCR no-template condition was non-specific amplification.

**Additional file 2. **Flow of synteny block search. Step 1: Conduct PSI-BLAST using CDS2 query and CDS4 query and deduplicated RefSeq proteins (‘gproteo_protein_uniq’ in Zenodo) as a database. Step 2: Retrieve the CDS information of RefSeq genome/replicon entries containing the CDS2 and CDS4 homologs in the gff format using R. We used get_seqid_paired_CDS.R. Step 3: Curate the result from step 2 manually to remove Int homologs occasionally detected as CDS2 hits and pseudogene products. List unwanted entries. Finalize the results in the gff format. Step 4: Retrieve the protein sequences of the CDS2 homolog and CDS4 homolog paired in one replicon. Construct multiple sequence alignment and obtain the distance matrix of CDS4 homologs in R. Decide the queries in the next round of PSI-BLAST. Step 5: Step 1–step 4 was repeated until the PSI-BLAST search converged or the most distant homolog from the query matched the query used in the previous round of PSI-BLAST.

**Additional file 3. **Full list of genomic locations of *tfp* and *srap* orthologs in the RefSeq files.

**Additional file 4. **RefSeq ID, Taxonomy ID, and presence of SE.

**Additional file 5. **Phylogenetic tree of SEs based on Tfp alignment. The color codes and symbols are identical to those in Fig. 6. The tree file and alignment file used are available in Figshare.

**Additional file 6. **The SE insertion and SE termini at 35 genomic locations.

**Additional file 7. **SEs carrying tmexCD-toprJ found in literatures.

**Additional file 8. **Coordinates, length, and GC percentage information of 37 SEs with identifiable termini. The number in the ID column corresponds to the panel number in the Additional file 6. The values in the region_start, region_end, and direction columns indicate the information used to generate the figure panels in Fig. 7 and Additional file 6. RefSeq IDs used for chromosomal GC percentage are shown in Sheet 2.

**Additional file 9. **Summary of amplicon sequencing of *attS*.

**Additional file 10. **Full list of genomic locations of *ginC* and *ginD* orthologs in the RefSeq files.

**Additional file 11. **Oligonucleotides used in this study.

## Data Availability

The dataset(s) supporting the conclusions of this article is available in the Zenodo. repository [doi:10.5281/zenodo.7839301 for Alphaproteobacteria protein dataset [83]; doi:10.5281/zenodo.5885688 for Betaproteobacteria protein dataset [84]; doi:10.5281/zenodo.5880327 for Gammaproteobacteria protein dataset [85]] and the Figshare repository [doi:10.6084/m9.figshare.19350761 for scripts, taxonomy data, alignments, tree files, Sanger sequencing chromatogram files, and the NGS data analysis methods [74]]. Raw sequence reads generated in this study are deposited to DDBJ Sequence Read Archive under accession numbers DRA013439 and DRA013440.

## Abbreviations

SE: Strand-biased circularizing integrative element
ICE: Integrative and conjugative element
IME: Integrative and mobilizable element
GInt: Genomic island with three integrases
RIT: Recombinase in trio
