# Supplementary material for: Host range of strand-biased circularizing integrative elements: a new class of mobile DNA elements nesting in Gammaproteobacteria
Source: Mob DNA. 2023 May 26;14:7. doi: 10.1186/s13100-023-00295-5 (PMC10214605; doi:10.1186/s13100-023-00295-5)
Supplement: Supplementary file 1 — Additional file 1. Results of the gene complementation test. Primer numbers correspond to the numbers in Fig. 2. (i) Strain BID1 harboring pBBR1MCS, (ii) strain BID1 harboring pBBR-intA, (iii) strain BID2 harboring pBBR1MCS, (iv) strain BID2 harboring pBBR-intB, (v) strain BID3 harboring pBBR1MCS, and (vi) strain BID3 harboring pBBR-srap. Electrophoresis was performed using a 2% agarose gel. The faint band in the attR PCR no-template condition was non-specific amplification. [file 13100_2023_295_MOESM1_ESM.pptx]

## Slide 1
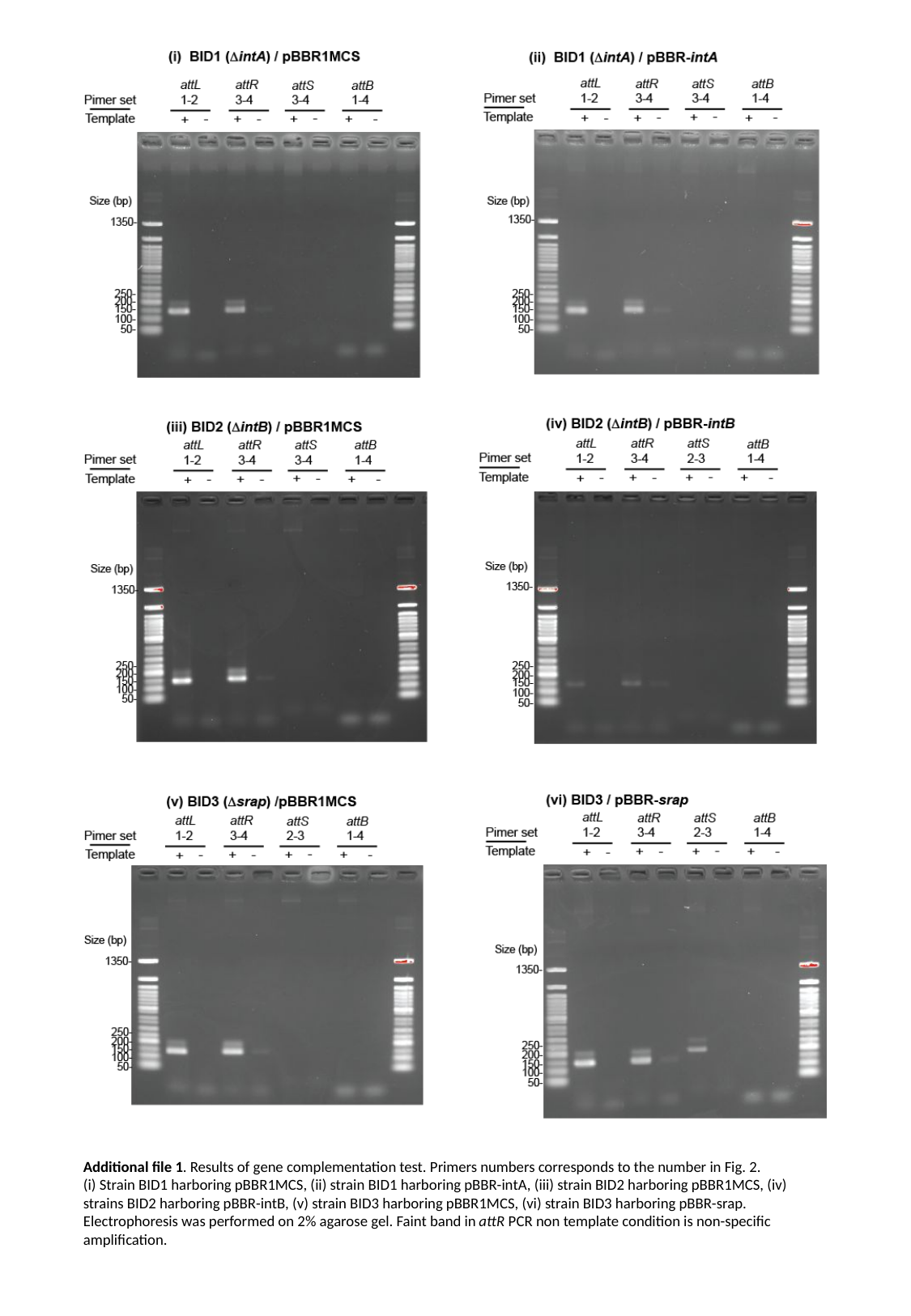

Additional file 1. Results of gene complementation test. Primers numbers corresponds to the number in Fig. 2.
(i) Strain BID1 harboring pBBR1MCS, (ii) strain BID1 harboring pBBR-intA, (iii) strain BID2 harboring pBBR1MCS, (iv) strains BID2 harboring pBBR-intB, (v) strain BID3 harboring pBBR1MCS, (vi) strain BID3 harboring pBBR-srap. Electrophoresis was performed on 2% agarose gel. Faint band in attR PCR non template condition is non-specific amplification.
