# Supplementary material for: Host range of strand-biased circularizing integrative elements: a new class of mobile DNA elements nesting in Gammaproteobacteria
Source: Mob DNA. 2023 May 26;14:7. doi: 10.1186/s13100-023-00295-5 (PMC10214605; doi:10.1186/s13100-023-00295-5)
Supplement: Supplementary file 2 — Additional file 2. Flow of synteny block search. Step 1: Conduct PSI-BLAST using CDS2 query and CDS4 query and deduplicated RefSeq proteins (‘gproteo_protein_uniq’ in Zenodo) as a database. Step 2: Retrieve the CDS information of RefSeq genome/replicon entries containing the CDS2 and CDS4 homologs in the gff format using R. We used get_seqid_paired_CDS.R. Step 3: Curate the result from step 2 manually to remove Int homologs occasionally detected as CDS2 hits and pseudogene products. List unwanted entries. Finalize the results in the gff format. Step 4: Retrieve the protein sequences of the CDS2 homolog and CDS4 homolog paired in one replicon. Construct multiple sequence alignment and obtain the distance matrix of CDS4 homologs in R. Decide the queries in the next round of PSI-BLAST. Step 5: Step 1–step 4 was repeated until the PSI-BLAST search converged or the most distant homolog from the query matched the query used in the previous round of PSI-BLAST. [file 13100_2023_295_MOESM2_ESM.pptx]

## Slide 1
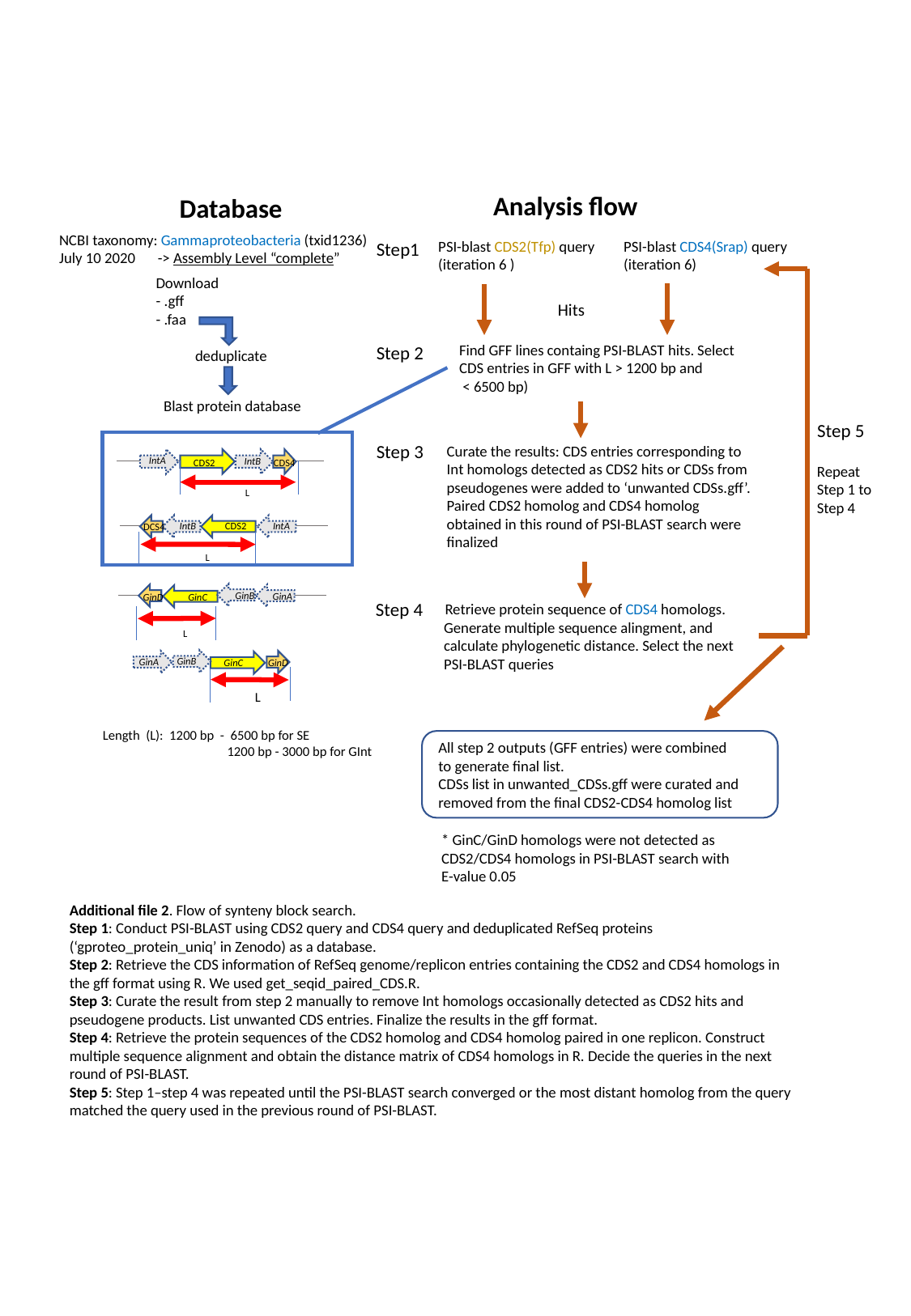

Analysis flow
Database
NCBI taxonomy: Gammaproteobacteria (txid1236)
July 10 2020　-> Assembly Level “complete”
PSI-blast CDS2(Tfp) query
(iteration 6 )
PSI-blast CDS4(Srap) query
(iteration 6)
Step1
Download
- .gff
- .faa
Hits
Find GFF lines containg PSI-BLAST hits. Select CDS entries in GFF with L > 1200 bp and
 < 6500 bp)
Step 2
deduplicate
Blast protein database
Step 5
Step 3
Curate the results: CDS entries corresponding to Int homologs detected as CDS2 hits or CDSs from pseudogenes were added to ‘unwanted CDSs.gff’. Paired CDS2 homolog and CDS4 homolog obtained in this round of PSI-BLAST search were finalized
IntA
IntB
CDS2
CDS4
Repeat Step 1 to Step 4
L
IntB
IntA
CDS2
DCS4
L
GinB
GinA
GinC
GinD
Step 4
Retrieve protein sequence of CDS4 homologs.
Generate multiple sequence alingment, and
calculate phylogenetic distance. Select the next PSI-BLAST queries
L
GinB
GinA
GinC
GinD
L
Length (L): 1200 bp - 6500 bp for SE
	 1200 bp - 3000 bp for GInt
All step 2 outputs (GFF entries) were combined
to generate final list.
CDSs list in unwanted_CDSs.gff were curated and
removed from the final CDS2-CDS4 homolog list
* GinC/GinD homologs were not detected as CDS2/CDS4 homologs in PSI-BLAST search with E-value 0.05
Additional file 2. Flow of synteny block search.
Step 1: Conduct PSI-BLAST using CDS2 query and CDS4 query and deduplicated RefSeq proteins (‘gproteo_protein_uniq’ in Zenodo) as a database.
Step 2: Retrieve the CDS information of RefSeq genome/replicon entries containing the CDS2 and CDS4 homologs in the gff format using R. We used get_seqid_paired_CDS.R.
Step 3: Curate the result from step 2 manually to remove Int homologs occasionally detected as CDS2 hits and pseudogene products. List unwanted CDS entries. Finalize the results in the gff format.
Step 4: Retrieve the protein sequences of the CDS2 homolog and CDS4 homolog paired in one replicon. Construct multiple sequence alignment and obtain the distance matrix of CDS4 homologs in R. Decide the queries in the next round of PSI-BLAST.
Step 5: Step 1–step 4 was repeated until the PSI-BLAST search converged or the most distant homolog from the query matched the query used in the previous round of PSI-BLAST.
