# Supplementary material for: Host range of strand-biased circularizing integrative elements: a new class of mobile DNA elements nesting in Gammaproteobacteria
Source: Mob DNA. 2023 May 26;14:7. doi: 10.1186/s13100-023-00295-5 (PMC10214605; doi:10.1186/s13100-023-00295-5)
Supplement: Supplementary file 5 — Additional file 5. Phylogenetic tree of SEs based on Tfp alignment. The color codes and symbols are identical to those in Fig. 6. The tree file and alignment file used are available in Figshare. [file 13100_2023_295_MOESM5_ESM.pptx]

## Slide 1
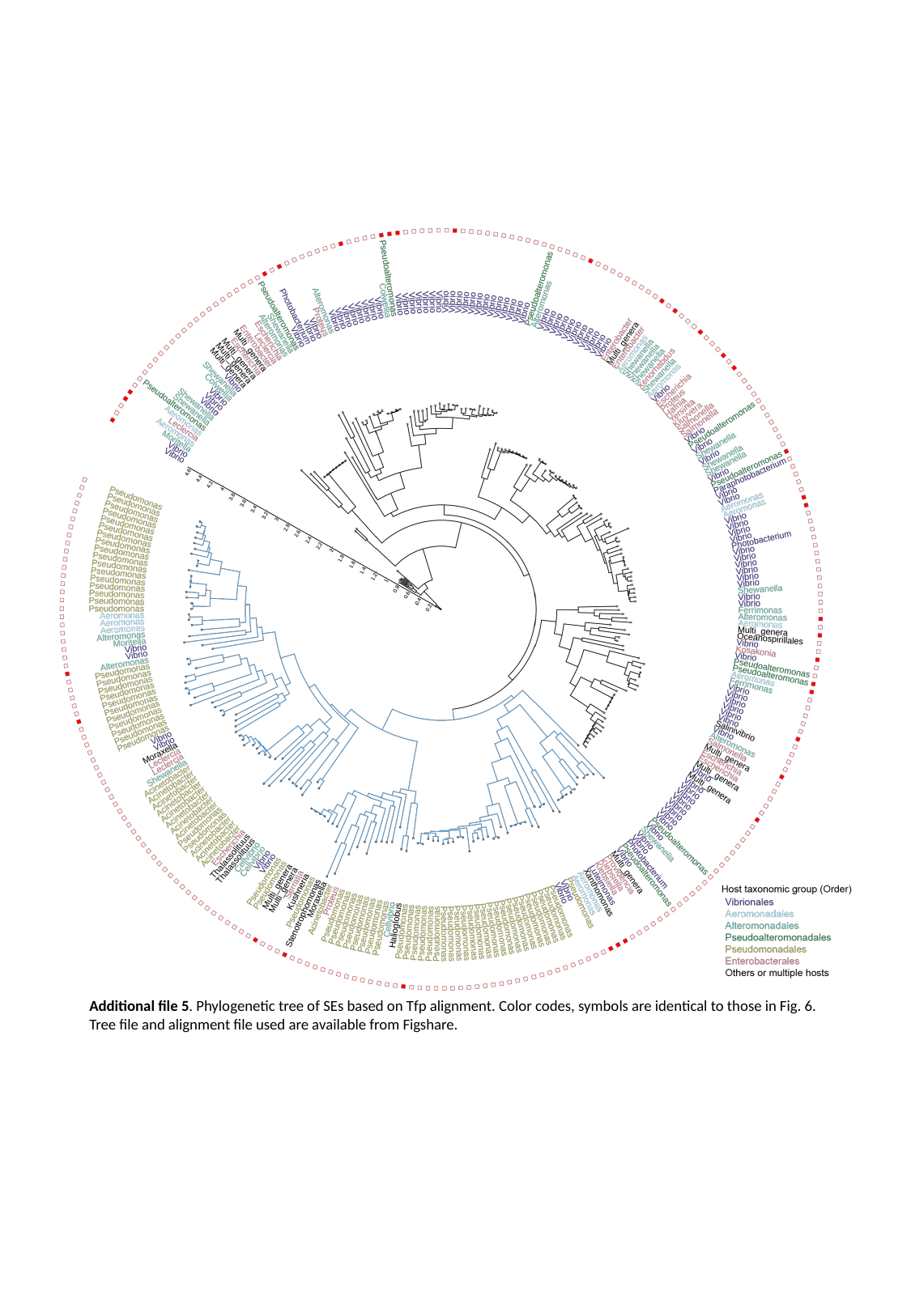

Additional file 5. Phylogenetic tree of SEs based on Tfp alignment. Color codes, symbols are identical to those in Fig. 6. Tree file and alignment file used are available from Figshare.
