# Supplementary material for: Host range of strand-biased circularizing integrative elements: a new class of mobile DNA elements nesting in Gammaproteobacteria
Source: Mob DNA. 2023 May 26;14:7. doi: 10.1186/s13100-023-00295-5 (PMC10214605; doi:10.1186/s13100-023-00295-5)

SEs with identifiable termini

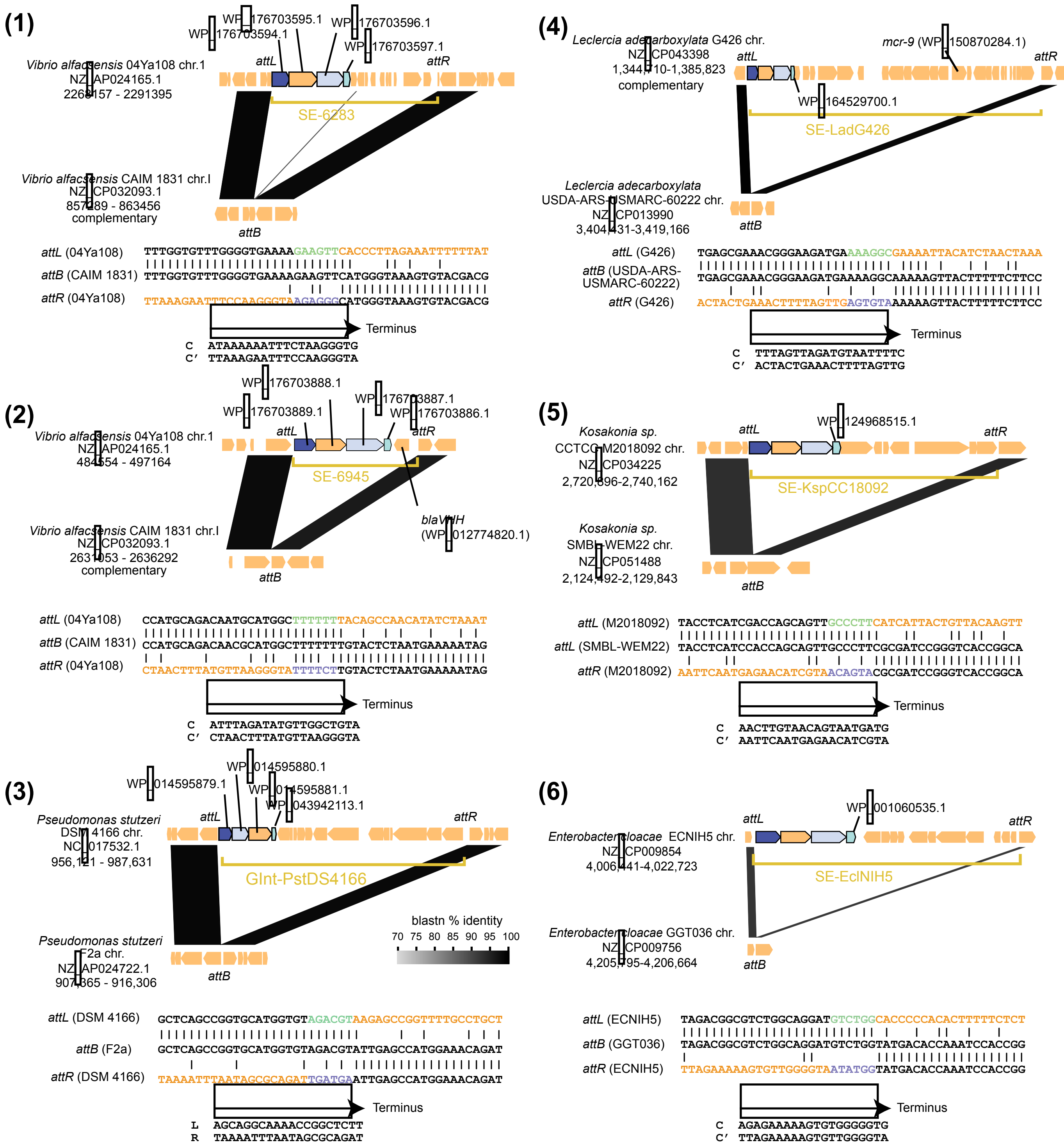

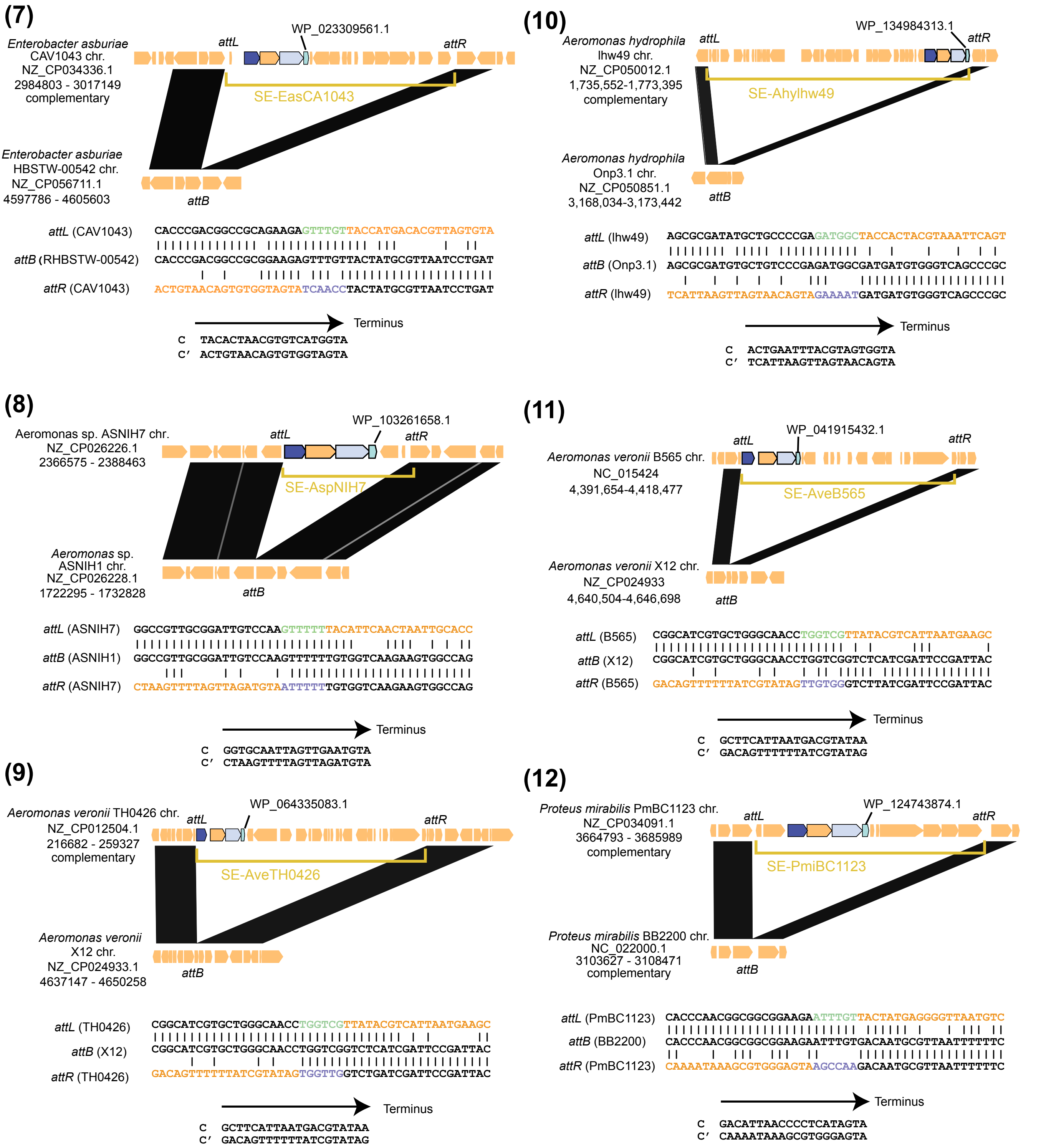

(7)
Enterobacter asburiae CAV1043 chr.
NZ_CP034336.1
2984803 - 3017149
complementary
attL
WP_023309561.1
attR
SE-EasCA1043
Enterobacter asburiae HBSTW-00542 chr.
NZ_CP056711.1
4597786 - 4605603
attB
attL (CAV1043) CACCCGACGGCCGCAGAAGAGTTTGTTACCATGACACGTTAGTGTA
attB (RHBSTW-00542) CACCCGACGGCCGCGGAAGAGTTTGTTACTATGCGTTAATCCTGAT
attR (CAV1043) ACTGTAACAGTGTGGTAGTATCAACCTACTATGCGTTAATCCTGAT
Terminus
C TACACTAACGTGTCATGGTA
C′ ACTGTAACAGTGTGGTAGTA
(10)
Aeromonas hydrophila lhw49 chr.
NZ_CP050012.1
1,735,552-1,773,395
complementary
attL
WP_134984313.1
attR
SE-Ahylhw49
Aeromonas hydrophila Onp3.1 chr.
NZ_CP050851.1
3,168,034-3,173,442
attB
attL (lhw49) AGCGCGATATGCTGCCCCGAGATGGCTACCACTACGTAAATTCAGT
attB (Onp3.1) AGCGCGATGTGCTGTCCCGAGATGGCGATGATGTGGGTCAGCCCGC
attR (lhw49) TCATTAAGTTAGTAACAGTAGAAAATGATGATGTGGGTCAGCCCGC
Terminus
C ACTGAATTTACGTAGTGGTA
C′ TCATTAAGTTAGTAACAGTA
(8)
Aeromonas sp. ASNIH7 chr.
NZ_CP026226.1
2366575 - 2388463
attL
WP_103261658.1
attR
SE-AspNIH7
Aeromonas sp. ASNIH1 chr.
NZ_CP026228.1
1722295 - 1732828
attB
attL (ASNIH7) GGCCGTTGCGGATTGTCCAAGTTTTTTACATTCAACTAATTGCACC
attB (ASNIH1) GGCCGTTGCGGATTGTCCAAGTTTTTTGTGGTCAAGAAGTGGCCAG
attR (ASNIH7) CTAAGTTTTAGTTAGATGTAATTTTTTGTGGTCAAGAAGTGGCCAG
Terminus
C GGTGCAATTAGTTGAATGTA
C′ CTAAGTTTTAGTTAGATGTA
(11)
Aeromonas veronii B565 chr.
NC_015424
4,391,654-4,418,477
attL
WP_041915432.1
attR
SE-AveB565
Aeromonas veronii X12 chr.
NZ_CP024933
4,640,504-4,646,698
attB
attL (B565) CGGCATCGTGCTGGGCAACCTGGTCGTTATACGTCATTAATGAAGC
attB (X12) CGGCATCGTGCTGGGCAACCTGGTCGGTCTCATCGATTCCGATTAC
attR (B565) GACAGTTTTTTATCGTATAGTTGTGGGTCTTATCGATTCCGATTAC
Terminus
C GCTTCATTAATGACGTATAA
C′ GACAGTTTTTTATCGTATAG
(9)
Aeromonas veronii TH0426 chr.
NZ_CP012504.1
216682 - 259327
complementary
attL
WP_064335083.1
attR
SE-AveTH0426
Aeromonas veronii X12 chr.
NZ_CP024933.1
4637147 - 4650258
attB
attL (TH0426) CGGCATCGTGCTGGGCAACCTGGTCGTTATACGTCATTAATGAAGC
attB (X12) CGGCATCGTGCTGGGCAACCTGGTCGGTCTCATCGATTCCGATTAC
attR (TH0426) GACAGTTTTTTATCGTATAGTGGTTGGTCTGATCGATTCCGATTAC
Terminus
C GCTTCATTAATGACGTATAA
C′ GACAGTTTTTTATCGTATAG
(12)
Proteus mirabilis PmBC1123 chr.
NZ_CP034091.1
3664793 - 3685989
complementary
attL
WP_124743874.1
attR
SE-PmiBC1123
Proteus mirabilis BB2200 chr.
NC_022000.1
3103627 - 3108471
complementary
attB
attL (PmBC1123) CACCCAACGGCGGCGGAAGAATTTGTTACTATGAGGGGTTAATGTC
attB (BB2200) CACCCAACGGCGGCGGAAGAATTTGTGACAATGCGTTAATTTTTTC
attR (PmBC1123) CAAAATAAAGCGTGGGAGTAAGCCAAGACAATGCGTTAATTTTTTC
Terminus
C GACATTAACCCCTCATAGTA
C′ CAAAATAAAGCGTGGGAGTA

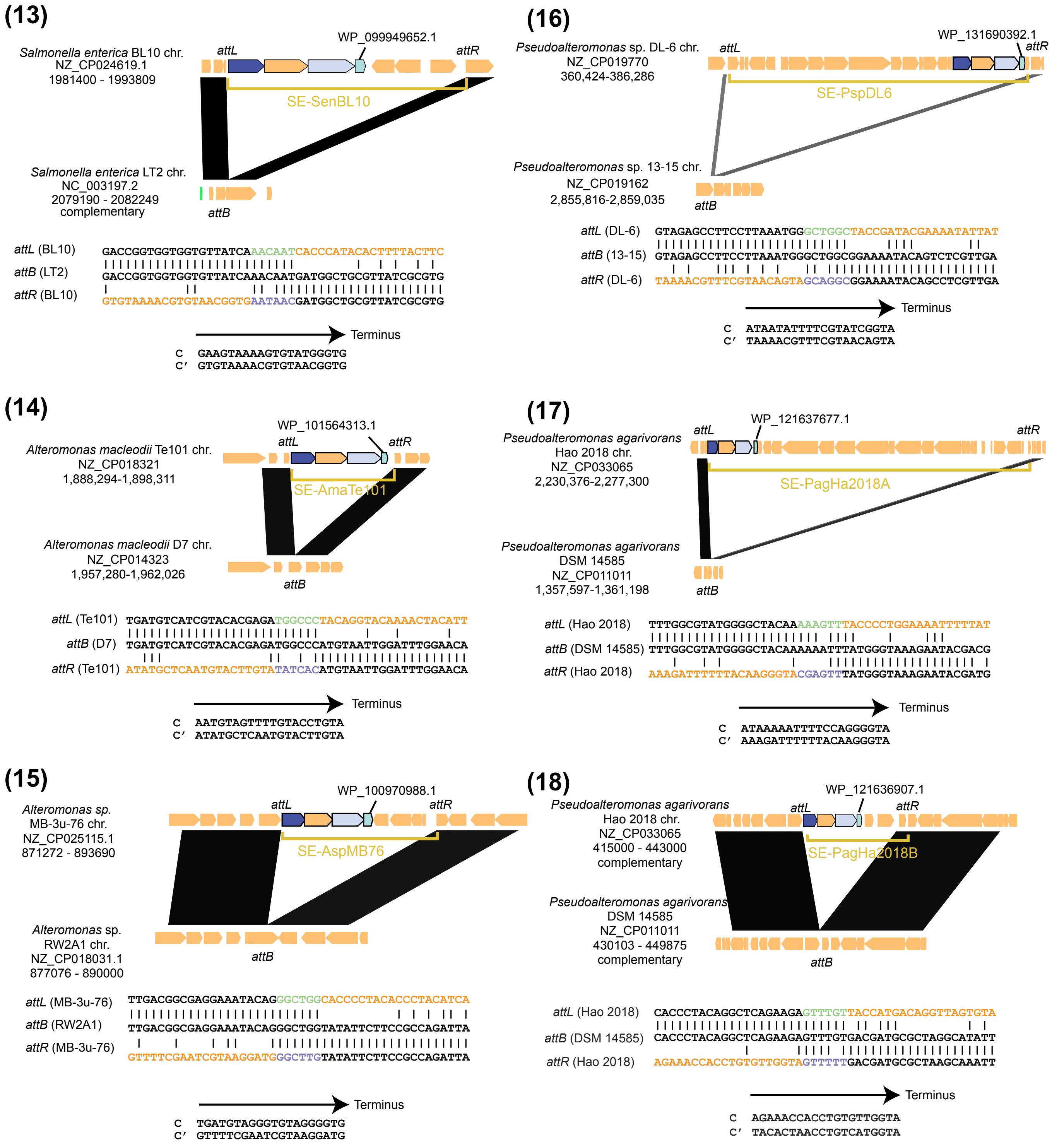
(13)
Salmonella enterica BL10 chr.
NZ_CP024619.1
1981400 - 1993809
attL
WP_099949652.1
attR
SE-SenBL10
Salmonella enterica LT2 chr.
NC_003197.2
2079190 - 2082249
complementary
attB
attL (BL10) GACCGGTGGTGGTGTTATCAAACAATCACCCATACACTTTTACTTC
attB (LT2) GACCGGTGGTGGTGTTATCAAACAATGATGGCTGCGTTATCGCGTG
attR (BL10) GTGTAAAACGTGTAACGGTGAATAACGATGGCTGCGTTATCGCGTG
Terminus
C GAAGTAAAAGTGTATGGGTG
C′ GTGTAAAACGTGTAACGGTG
(16)
Pseudoalteromonas sp. DL-6 chr.
NZ_CP019770
360,424-386,286
attL
WP_131690392.1
attR
SE-PspDL6
Pseudoalteromonas sp. 13-15 chr.
NZ_CP019162
2,855,816-2,859,035
attB
attL (DL-6) GTAGAGCCTTCCTTAAATGGGCTGGCTACCGATACGAAAATATTAT
attB (13-15) GTAGAGCCTTCCTTAAATGGGCTGGCGGAAAATACAGTCTCGTTGA
attR (DL-6) TAAAACGTTTCGTAACAGTAGCAGGCGGAAAATACAGCCTCGTTGA
Terminus
C ATAATATTTTCGTATCGGTA
C′ TAAAACGTTTCGTAACAGTA
(14)
Alteromonas macleodii Te101 chr.
NZ_CP018321
1,888,294-1,898,311
WP_101564313.1
attL
attR
SE-AmaTe101
Alteromonas macleodii D7 chr.
NZ_CP014323
1,957,280-1,962,026
attB
attL (Te101) TGATGTCATCGTACACGAGATGGCCCTACAGGTACAAAACTACATT
attB (D7) TGATGTCATCGTACACGAGATGGCCCATGTAATTGGATTTGGAACA
attR (Te101) ATATGCTCAATGTACTTGTATATCACATGTAATTGGATTTGGAACA
Terminus
C AATGTAGTTTTGTACCTGTA
C′ ATATGCTCAATGTACTTGTA
(17)
Pseudoalteromonas agarivorans
Hao 2018 chr.
NZ_CP033065
2,230,376-2,277,300
attL
WP_121637677.1
attR
SE-PagHa2018A
Pseudoalteromonas agarivorans
DSM 14585
NZ_CP011011
1,357,597-1,361,198
attB
attL (Hao 2018) TTTGGCGTATGGGGCTACAAAAAGTTTACCCCTGGAAAATTTTTAT
attB (DSM 14585) TTTGGCGTATGGGGCTACAAAAAATTTATGGGTAAAGAATACGACG
attR (Hao 2018) AAAGATTTTTTACAAGGGTACGAGTTTATGGGTAAAGAATACGATG
Terminus
C ATAAAAATTTTCCAGGGGTA
C′ AAAGATTTTTTACAAGGGTA
(15)
Alteromonas sp.
MB-3u-76 chr.
NZ_CP025115.1
871272 - 893690
WP_100970988.1
attL
attR
SE-AspMB76
Alteromonas sp.
RW2A1 chr.
NZ_CP018031.1
877076 - 890000
attB
attL (MB-3u-76) TTGACGGCGAGGAAATACAGGGCTGGCACCCCTACACCCTACATCA
attB (RW2A1) TTGACGGCGAGGAAATACAGGGCTGGTATATTCTTCCGCCAGATTA
attR (MB-3u-76) GTTTTCGAATCGTAAGGATGGGCTTGTATATTCTTCCGCCAGATTA
Terminus
C TGATGTAGGGTGTAGGGGTG
C′ GTTTTCGAATCGTAAGGATG
(18)
Pseudoalteromonas agarivorans
Hao 2018 chr.
NZ_CP033065
415000 - 443000
complementary
WP_121636907.1
attL
attR
SE-PagHa2018B
Pseudoalteromonas agarivorans
DSM 14585
NZ_CP011011
430103 - 449875
complementary
attB
attL (Hao 2018) CACCCTACAGGCTCAGAAGAGTTTGTTACCATGACAGGTTAGTGTA
attB (DSM 14585) CACCCTACAGGCTCAGAAGAGTTTGTGACGATGCGCTAGGCATATT
attR (Hao 2018) AGAAACCACCTGTGTTGGTAGTTTTTGACGATGCGCTAAGCAAATT
Terminus
C AGAAACCACCTGTGTTGGTA
C′ TACACTAACCTGTCATGGTA

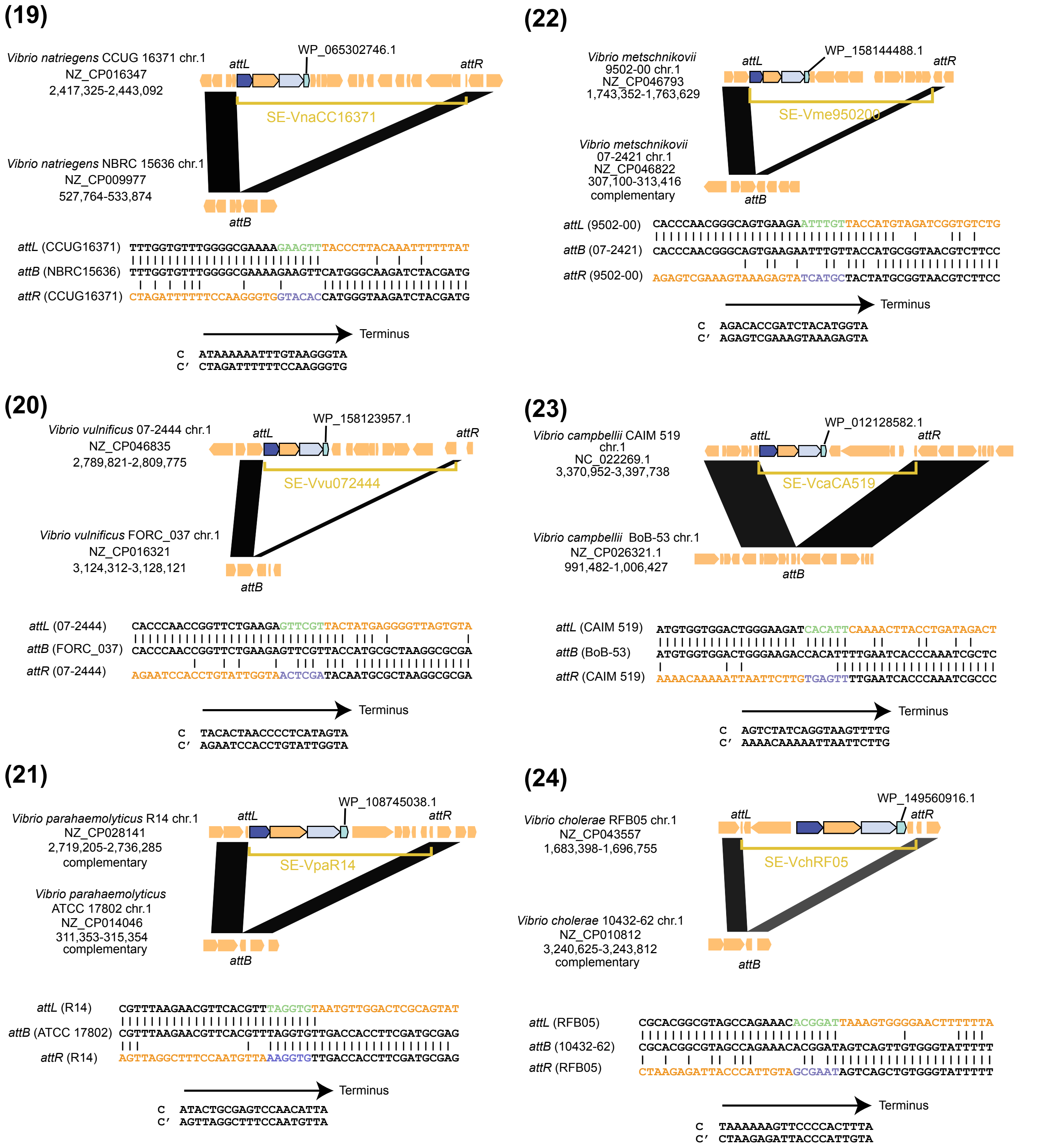

(19)
Vibrio natriegens CCUG 16371 chr.1
NZ_CP016347
2,417,325-2,443,092
attL
WP_065302746.1
attR
SE-VnaCC16371
Vibrio natriegens NBRC 15636 chr.1
NZ_CP009977
527,764-533,874
attB
attL (CCUG16371) TTTGGTGTTTGGGGCGAAAAGAAGTTTACCCTTACAAATTTTTTAT
attB (NBRC15636) TTTGGTGTTTGGGGCGAAAAGAAGTTCATGGGCAAGATCTACGATG
attR (CCUG16371) CTAGATTTTTTCCAAGGGTGGTACACCATGGGTAAGATCTACGATG
Terminus
C ATAAAAAATTTGTAAGGGTA
C′ CTAGATTTTTTCCAAGGGTG
(22)
Vibrio metschnikovii
9502-00 chr.1
NZ_CP046793
1,743,352-1,763,629
attL
WP_158144488.1
attR
SE-Vme950200
Vibrio metschnikovii
07-2421 chr.1
NZ_CP046822
307,100-313,416
complementary
attB
attL (9502-00) CACCCAACGGGCAGTGAAGAATTTGTTACCATGTAGATCGGTGTCTG
attB (07-2421) CACCCAACGGGCAGTGAAGAATTTGTTACCATGCGGTAACGTCTTCC
attR (9502-00) AGAGTCGAAAGTAAAGAGTATCATGCTACTATGCGGTAACGTCTTCC
Terminus
C AGACACCGATCTACATGGTA
C′ AGAGTCGAAAGTAAAGAGTA
(20)
Vibrio vulnificus 07-2444 chr.1
NZ_CP046835
2,789,821-2,809,775
attL
WP_158123957.1
attR
SE-Vvu072444
Vibrio vulnificus FORC_037 chr.1
NZ_CP016321
3,124,312-3,128,121
attB
attL (07-2444) CACCCAACCGGTTCTGAAGAGTTCGTTACTATGAGGGGTTAGTGTA
attB (FORC_037) CACCCAACCGGTTCTGAAGAGTTCGTTACCATGCGCTAAGGCGCGA
attR (07-2444) AGAATCCACCTGTATTGGTAACTCGATACAATGCGCTAAGGCGCGA
Terminus
C TACACTAACCCCTCATAGTA
C′ AGAATCCACCTGTATTGGTA
(23)
Vibrio campbellii CAIM 519
chr.1
NC_022269.1
3,370,952-3,397,738
attL
WP_012128582.1
attR
SE-VcaCA519
Vibrio campbellii BoB-53 chr.1
NZ_CP026321.1
991,482-1,006,427
attB
attL (CAIM 519) ATGTGGTGGACTGGGAAGATCACATTCAAAACTTACCTGATAGACT
attB (BoB-53) ATGTGGTGGACTGGGAAGACCACATTTGAATCACCCAAATCGCTC
attR (CAIM 519) AAAACAAAAATTAATTCTTGTGAGTTTTGAATCACCCAAATCGCCC
Terminus
C AGTCTATCAGGTAAGTTTTG
C′ AAAACAAAAATTAATTCTTG
(21)
Vibrio parahaemolyticus R14 chr.1
NZ_CP028141
2,719,205-2,736,285
complementary
attL
WP_108745038.1
attR
SE-VpaR14
Vibrio parahaemolyticus
ATCC 17802 chr.1
NZ_CP014046
311,353-315,354
complementary
attB
attL (R14) CGTTTAAGAACGTTCACGTTTAGGTGTAATGTTGGACTCGCAGTAT
attB (ATCC 17802) CGTTTAAGAACGTTCACGTTTAGGTGTTGACCACCTTCGATGCGAG
attR (R14) AGTTAGGCTTTCCAATGTTAAAGGTGTTGACCACCTTCGATGCGAG
Terminus
C ATACTGCGAGTCCAACATTA
C′ AGTTAGGCTTTCCAATGTTA
(24)
Vibrio cholerae RFB05 chr.1
NZ_CP043557
1,683,398-1,696,755
attL
WP_149560916.1
attR
SE-VchRF05
Vibrio cholerae 10432-62 chr.1
NZ_CP010812
3,240,625-3,243,812
complementary
attB
attL (RFB05) CGCACGGCGTAGCCAGAAACACGGATTAAAGTGGGGAACTTTTTTA
attB (10432-62) CGCACGGCGTAGCCAGAAACACGGATAGTCAGTTGTGGGTATTTTT
attR (RFB05) CTAAGAGATTACCCATTGTAGCGAATAGTCAGCTGTGGGTATTTTT
Terminus
C TAAAAAAGTTCCCCACTTTA
C′ CTAAGAGATTACCCATTGTA

(25)

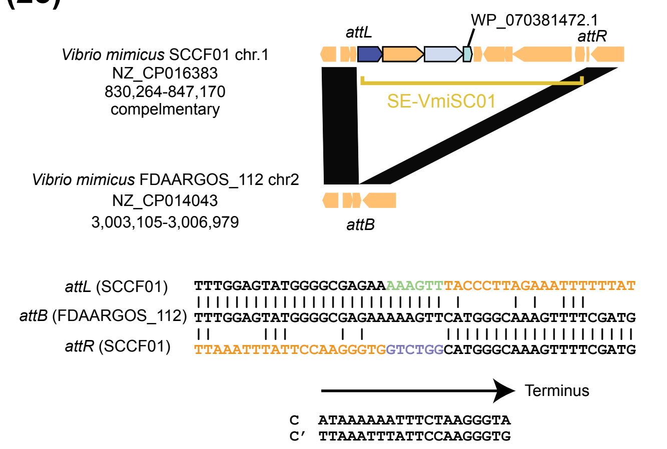

(28)

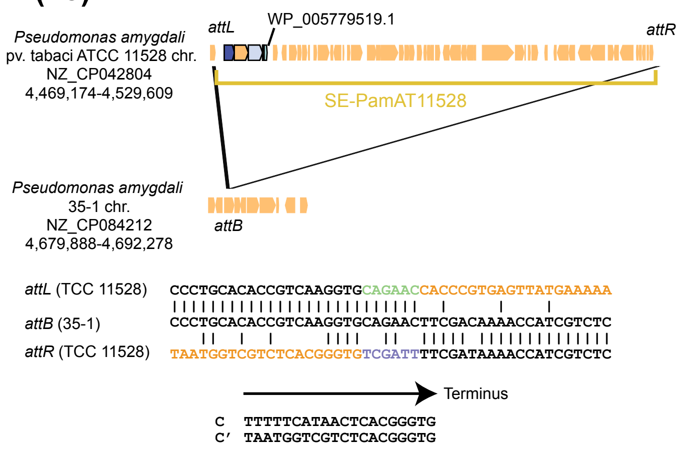

(26)

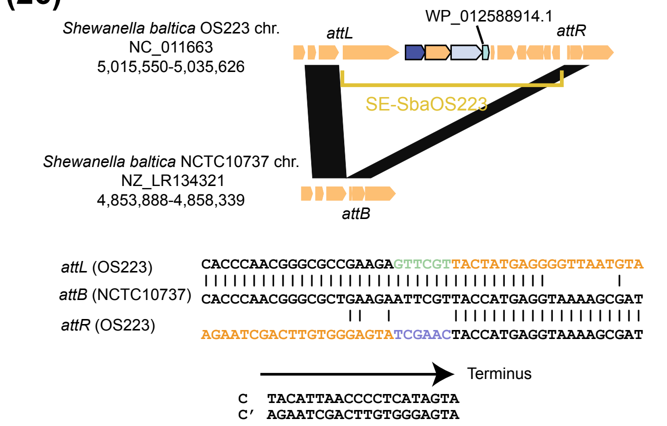

(29)

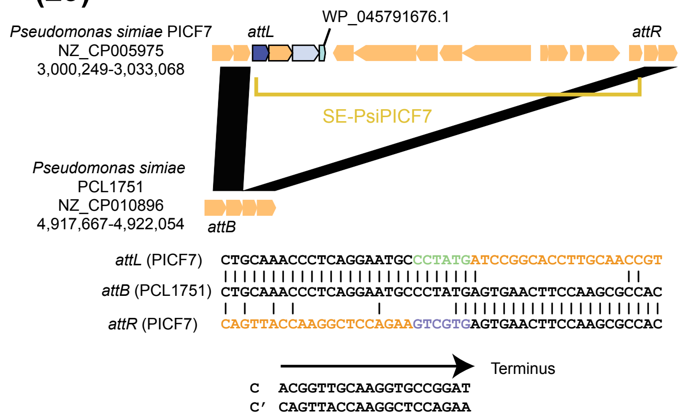

(27)

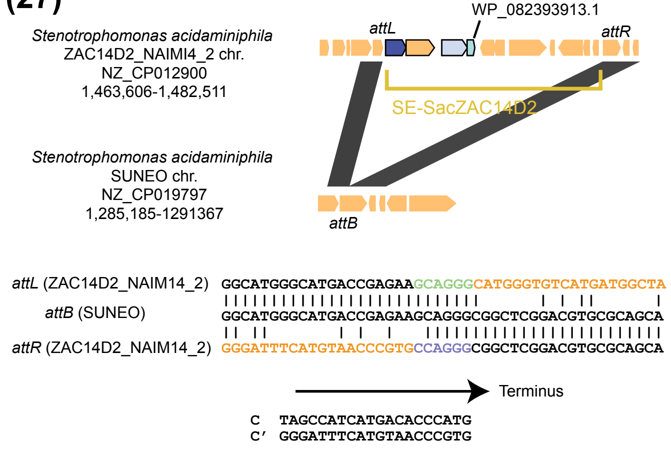

(30)

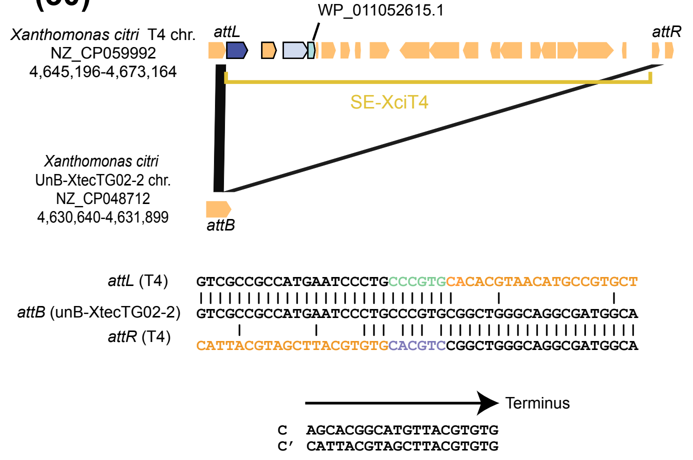

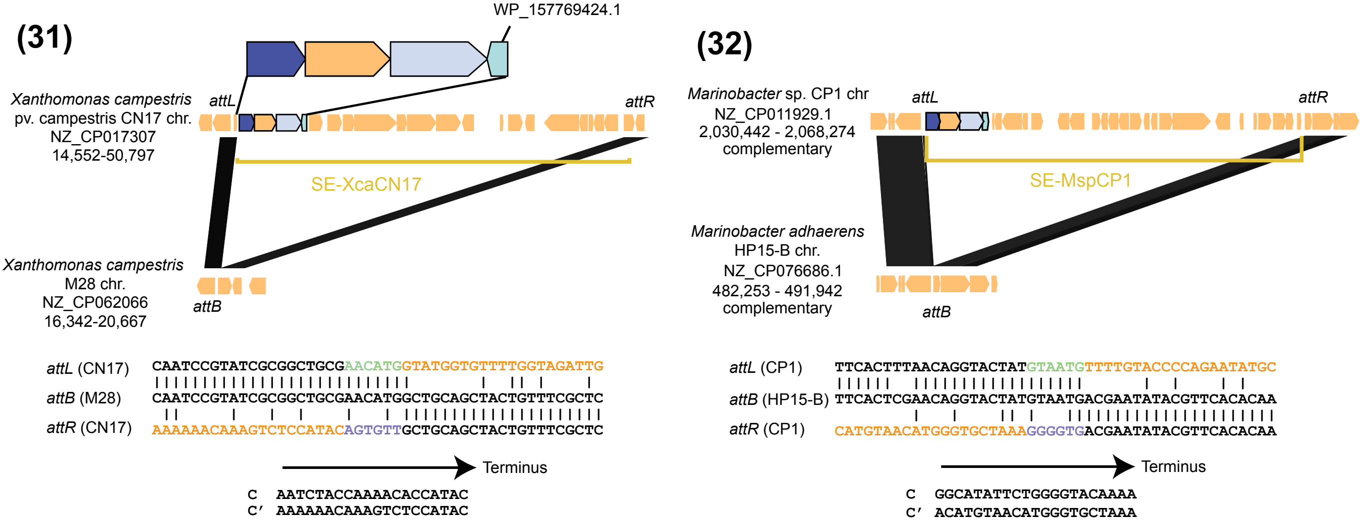

SE core genes embedded in a plasticity region

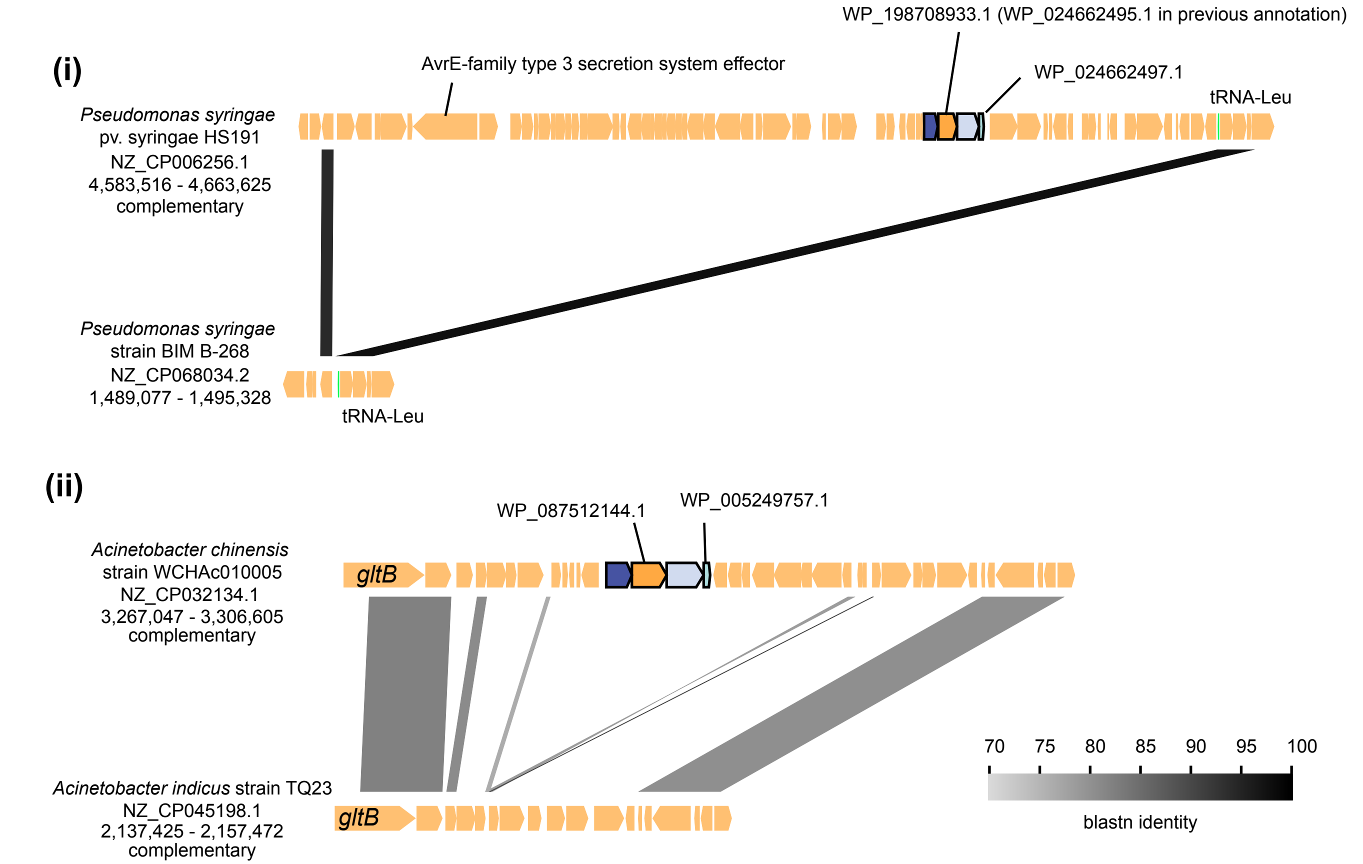

Supplement: Supplementary file 6 — Additional file 6. The SE insertion and SE termini at 35 genomic locations. [file 13100_2023_295_MOESM6_ESM.pdf]
