# Supplementary figures and images for: Host range of strand-biased circularizing integrative elements: a new class of mobile DNA elements nesting in Gammaproteobacteria
Source: Mob DNA. 2023 May 26;14:7. doi: 10.1186/s13100-023-00295-5 (PMC10214605; doi:10.1186/s13100-023-00295-5)

## Slide 1
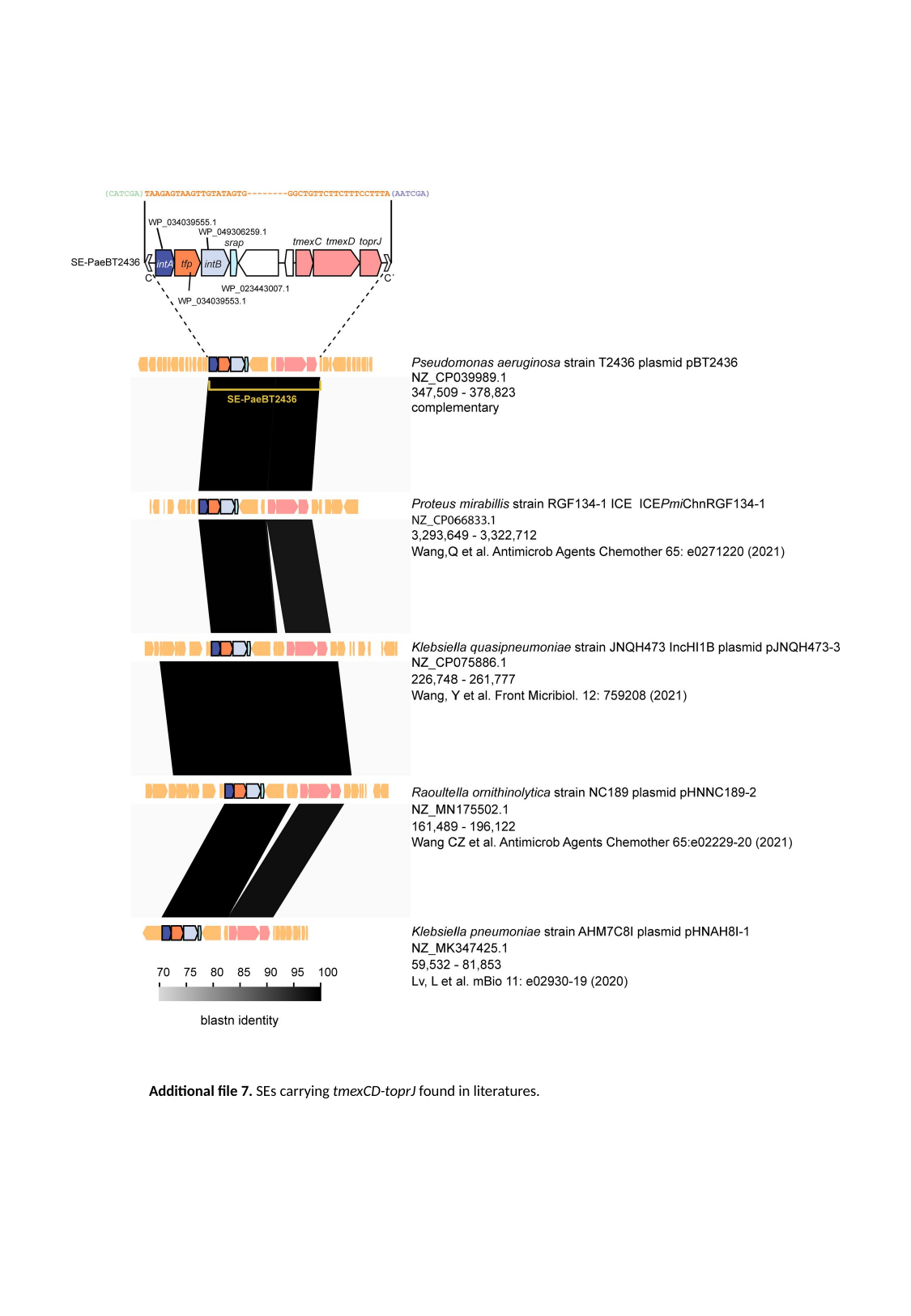

Additional file 7. SEs carrying tmexCD-toprJ found in literatures.

Supplement: Supplementary file 7 — Additional file 7. SEs carrying tmexCD-toprJ found in literatures. [file 13100_2023_295_MOESM7_ESM.pptx]
